# Cation Influence on Hot-Carrier Relaxation in Tin Triiodide Perovskite Thin Films

**DOI:** 10.1021/acsenergylett.4c00055

**Published:** 2024-02-15

**Authors:** Larissa J. M. van de Ven, Eelco K. Tekelenburg, Matteo Pitaro, Jacopo Pinna, Maria A. Loi

**Affiliations:** Zernike Institute for Advanced Materials, University of Groningen, Nijenborgh 4, 9747 AG Groningen, The Netherlands

## Abstract

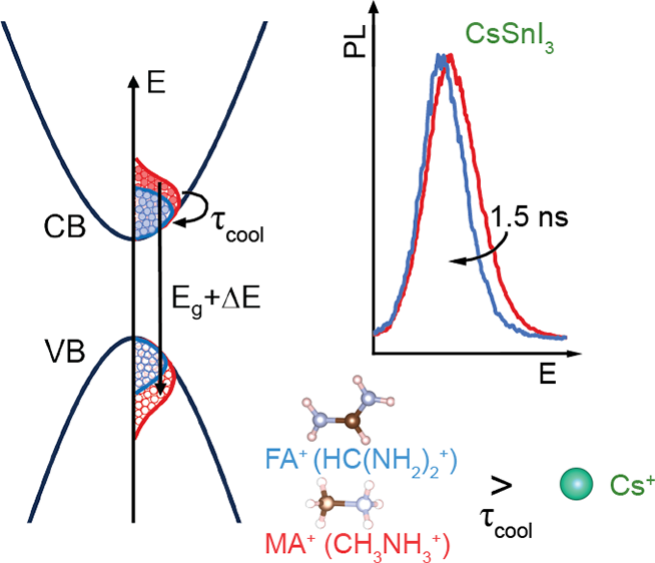

Slow hot-carrier cooling may potentially allow overcoming the maximum achievable power conversion efficiency of single-junction solar cells. For formamidinium tin triiodide, an exceptional slow cooling time of a few nanoseconds was reported. However, a systematic study of the cation influence, as is present for lead compounds, is lacking. Here, we report the first comparative study on formamidinium, methylammonium, and cesium tin triiodide thin films. By investigating their photoluminescence, we observe a considerable shift of the emission peak to high energy with the increase of the excited-state population, which is more prominent in the case of the two hybrid organic–inorganic perovskites (∼45 meV vs ∼15 meV at 9 × 10^17^ cm^–3^ carrier density). The hot-carrier photoluminescence of the three tin compositions decays with a 0.6–2.8 ns time constant with slower cooling observed for the two hybrids, further indicating their importance.

Due to the outstanding optoelectronic properties of metal halide perovskites, there has been a surge in research effort for their use as active materials in solar cells, leading to an unprecedented increase in power conversion efficiency from 3.8% in 2009 to 26.1% in 2023.^1,2^ However, the efficiency of conventional metal halide single-junction solar cells appears to be close to saturation as it is approaching the detailed balance limit.^3^ The rapid relaxation of above-band gap photoexcited carriers, i.e., hot carriers, via heat dissipation is a major energy loss channel in photovoltaics and largely contributes to this limit.^3,4^ A sufficiently retarded hot-carrier cooling time, leading to a long-lived hot-carrier population, could allow for their extraction, circumventing energy losses related to the cooling. This is the principle behind a hot-carrier solar cell that could theoretically push the power conversion efficiency limit to 66%.^5^ While in typical semiconductors, such as GaAs, cooling occurs within 1 ps, significantly slower cooling times have been reported for metal halide perovskites.^6−12^

For lead-based perovskites cooling times of several tens to several hundreds of picoseconds have been found.^6−11^ Furthermore, several reports on lead-based perovskites indicate that the A-site cation plays a role in the cooling dynamics.^6,7,9,13^ Yang et al. reported a similar cooling time constant for MAPbI_3_ and FAPbI_3_ of about 30 ps at a carrier density of ∼6 × 10^18^ cm^–3^.^6^ Yang et al. showed that the all-inorganic cesium-based perovskite, on the other hand, has a relaxation lifetime 10-fold faster than that of FAPbI_3_ (37 ps vs 305 ps after *T*_c_ = 400 K at ∼2 × 10^18^ cm^–3^).^9^ This is in line with the findings of Zhu et al. on lead bromide single crystals, where a similar cooling time constant of 150 ± 30 ps and 190 ± 20 ps in the initial cooling stage was found for MAPbBr_3_ and FAPbBr_3_, respectively, but no effect in CsPbBr_3_ was observed, suggesting that it cooled within their experimental resolution (20 ps).^7^ However, Hopper et al. claimed the opposite by reporting a cooling time constant of 0.8 ps for CsPbBr_3_ and about 0.4 ps for the hybrids at ∼2 × 10^18^ cm^–3^, with a stronger dependence of this time constant with the carrier density for CsPbBr_3_.^13^

In 2018, an exceptional slow hot-carrier cooling (a few ns) in formamidinium (HC(NH_2_)_2_^+^) tin triiodide (FASnI_3_) was reported.^11^ This raises the question of whether other tin triiodide perovskites also exhibit slow hot-carrier cooling and whether there is an influence on the nature of the cation. However, such a comparative study, as described above for the lead compounds, is still lacking in tin-based perovskites. Here, we aim to bridge this gap by studying cesium tin triiodide (CsSnI_3_) and methylammonium (CH_3_NH_3_^+^) tin triiodide (MASnI_3_) alongside FASnI_3_.

One of the reasons for this gap in the literature is the difficulty in producing high-quality films of the three tin compounds and the need to independently optimize each sample processing condition. Hurdles in obtaining high-quality, stable tin perovskite thin films are the low formation energies of Sn-vacancy defects and the ease of oxidation of Sn^2+^ to Sn^4+^ that result in reduced stability and a high density of background holes.^14−16^ The importance of the material quality lies in the fact that it has been shown to have a profound impact on the optical properties of tin metal halide perovskites, including on the relaxation time of hot carriers.^6,9,17−20^ It is found that hot carriers can specifically efficiently couple with defects, providing additional cooling pathways through defect states and that a higher density of “cold” background carriers may offer rapid thermalization pathways through carrier–carrier scattering.^9,18−22^ This is why prior to investigating the hot-carrier cooling dynamics, we present our successful efforts in improving the thin-film quality of CsSnI_3_ and MASnI_3_.

Through the use of energy-resolved and time-resolved photoluminescence (PL) spectroscopy, we show for the first time that slow hot-carrier cooling in tin perovskites is not only limited to FASnI_3_ but both MASnI_3_ and CsSnI_3_ exhibit the phenomenon as well. Within our described optimization of the sample quality, the two hybrids show a stronger hot-carrier contribution and a slower decay thereof with time constants of 2.0 and 2.8 ns. Importantly, this exceeds the reported necessary time of 1 ns that is expected to promote appreciable efficiency increases.^23^

At first, we made an effort to improve the film quality of CsSnI_3_ and MASnI_3_ to get them to a quality similar to that of our FASnI_3_ films. In this endeavor, we modified, among other parameters, the solvent, the antisolvent, the concentration of the solution, the deposition timing, and the annealing procedure. After each optimization step, key performance indicators comprising intense and narrow PL and a long PL lifetime were considered, since together they signify a reduced defect density.^18^ Minute substitution (8 mol %) of FAI by phenylethylammonium iodide (PEAI), to obtain so-called 2D/3D thin films, has been shown to lead to smoothened grain boundaries, better coverage, and extended ordering of the crystal planes, thereby suppressing trap-assisted recombination without compromising the desirable 3D perovskite properties of the thin film.^24^ This served as an incentive to explore the PEAI substitution for both CsSnI_3_ and MASnI_3_ prepared under the final processing conditions. The preparation details of the films made with the starting recipe (same conditions as for FASnI_3_),^24^ the improved recipe, and the 2D/3D recipe are described in the Supporting Information.

Our meticulous optimization strategy results in substantial improvement of the PL characteristics of CsSnI_3_, as reported in Figure 1a,b. Interestingly, by varying just the processing conditions, i.e., without any PEAI substitution, we observe an improved PL intensity and lifetime. For MASnI_3_ this improvement is more prominent with about a 6-fold increase in PL intensity and an increased lifetime from hundreds of picoseconds to several nanoseconds, indicating an effective suppression of trap-assisted recombination (Figures 1c and S1). In addition, the improved MASnI_3_ film displays smaller pinholes, smoother grain boundaries, and a reduced polydispersity of the grains, leading to smooth films (SEM in Figure 1f,g; AFM in Figure S2).

**Figure 1:**
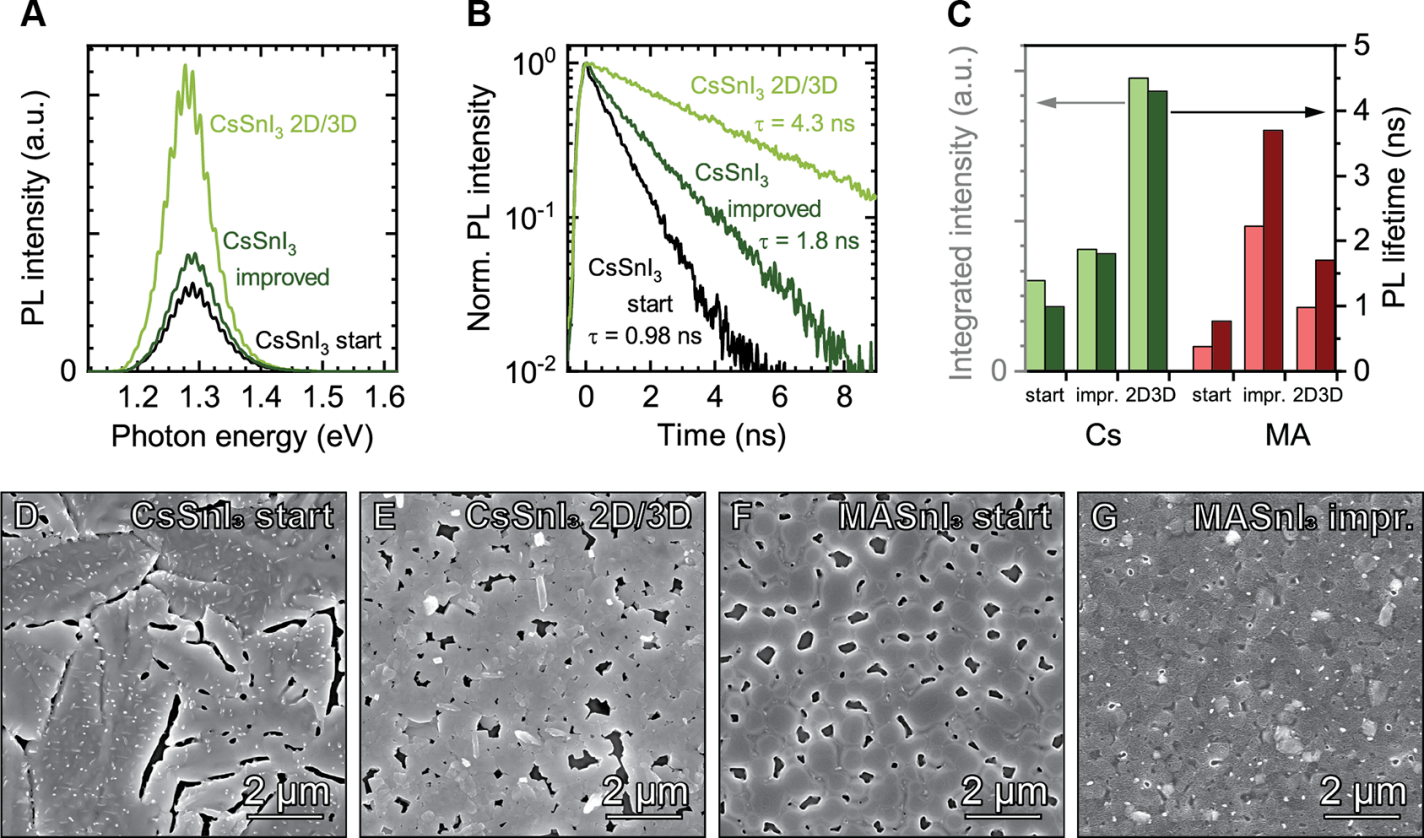
(a) Energy-resolved PL and (b) time-resolved PL over the course of the improvements in CsSnI_3_, i.e., going from the starting recipe to the improved recipe to the 2D/3D recipe. (c) Overview of the improvements in integrated PL intensity and PL lifetime for CsSnI_3_ and MASnI_3_. (d–g) SEM micrographs of starting CsSnI_3_, 2D/3D CsSnI_3_, starting MASnI_3_, and MASnI_3_ with improved processing conditions on glass substrates.

By using the 2D/3D substitution method, we were able to improve the thin-film quality of FASnI_3_; 2D/3D FASnI_3_ results in an improved PL lifetime of τ = 7.5 ns at 25 nJ cm^–2^, a higher degree of crystallinity, and a more compact, smooth film (Figures S3 and S4). These results are in good agreement with previous work of our group.^24^ For both CsSnI_3_ and MASnI_3_, minute PEA substitution results in (further) smoothening of grain boundaries and better film coverage compared to the pure 3D counterpart (Figures 1d,e, S2, and S5). We note that the films in Figure 1e,g still exhibit no full coverage. This is a commonly recognized challenge—even in high-quality tin-based perovskites—that is caused by the strong Lewis acidity of Sn^2+^ leading to a fast, uncontrolled crystallization.^25−30^ We do acknowledge that full coverage is key in obtaining solar cells with low shunt resistance, and it has been achieved in a few reports;^31−33^ however, solar cell fabrication is not our aim.

Apart from the morphological improvements, we investigated the effects of the 2D/3D method on the crystallinity. While no improvements in the already highly crystalline MASnI_3_ film is obtained through PEA substitution (Figure S3), in CsSnI_3_ the PEA substitution results in higher crystallinity as signified by narrow XRD peaks with increased signal-to-noise ratio (Figure S6).

As a consequence, the PL lifetime improves further from τ = 1.8 ns to τ = 4.3 ns for 2D/3D CsSnI_3_ (Figure 1b). In addition, the CsSnI_3_ 2D/3D film shows enhanced PL intensity with a concomitant narrowing of the PL fwhm and a small red-shift (Figure 1a), which we attribute to reduced defect and doping density, respectively.^17,18,34,35^ For MASnI_3_ on the other hand, the 2D/3D film shows a reduced PL intensity and lifetime and an increased fwhm compared to those of the 3D film with optimized processing parameters (Figures 1c and S1).

We propose that the minute substitution of PEA for MA has a nontrivial effect on the crystallization of the thin films. To further investigate this suggestion, we performed PL measurements on both the front (perovskite surface) and back (perovskite/glass interface) of all samples, as shown in Figure S7. For 2D/3D FASnI_3_ and 2D/3D CsSnI_3_, the PL signal stemming from low-dimensional perovskite phases (PEA_2_A_*n*–1_Sn_*n*_I_3*n*+1_) is only significantly detected at the back, showing that their presence is limited to the perovskite/glass interface. In contrast, for 2D/3D MASnI_3_ the PL signal from low-dimensional phases is detected from both sides, showing their presence throughout the bulk, which we propose to negatively impact the order of the film.

Since material quality plays a role in extending the hot-carrier cooling time, the highest quality films of all compounds, i.e., 2D/3D for FASnI_3_ and CsSnI_3_ and optimized 3D for MASnI_3_, are investigated in the remaining part of the study and are denoted by their chemical formula only for brevity.^6,9,18,20^ The optical properties and crystal structure of these films are summarized in Figure 2. Figure 2a shows the normalized photoluminescence and absorbance spectra of all three compounds. The PL peaks exhibit a progressive redshift when exchanging FA with Cs to MA (1.37 eV, 1.28 eV, and 1.25 eV, respectively). Via Tauc plots the band gaps of FASnI_3_, CsSnI_3_, and MASnI_3_ are estimated to be 1.38, 1.29, and 1.28 eV, respectively, meaning that all compounds exhibit small Stokes shifts (Figure S8).

**Figure 2:**
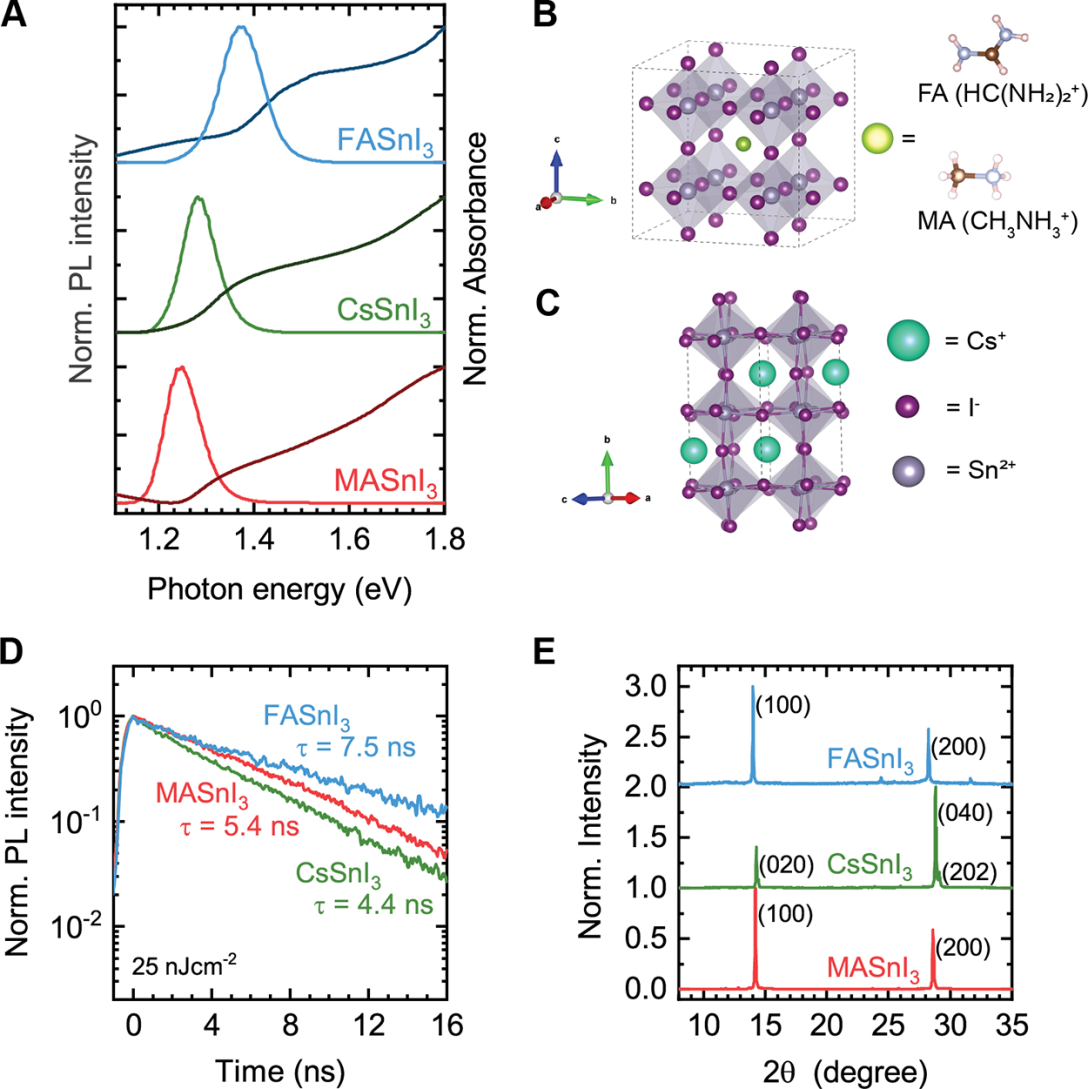
(a) Normalized photoluminescence and normalized (at 1.8 eV) absorbance spectra of FASnI_3_, CsSnI_3_, and MASnI_3_. (b) Representation of the cubic crystal structure of FASnI_3_ and MASnI_3_, where the organic cations are rotationally disordered and (c) of the orthorhombic crystal structure of CsSnI_3_. Generated from reported structures in refs (36 and 37). (d) PL lifetime and (e) XRD patterns for all three compounds. The crystal planes are indicated for the cubic *Pm*3̅*m* and orthorhombic *Pnma* crystal structures of the hybrid and the inorganic perovskites, respectively.

The redshift can be readily explained considering their crystal structures, as displayed in Figure 2b,c. Both FASnI_3_ and MASnI_3_ have a cubic crystal structure, where the larger ionic radius of formamidinium (1.9–2.2 Å) with respect to methylammonium (1.8 Å) extends the Sn–I bond length.^37−40^ This extension leads to a reduced orbital overlap at the band edges that subsequently explains the larger band gap for FASnI_3_. CsSnI_3_, on the other hand, crystallizes in an orthorhombic crystal structure as the small ionic radius of cesium (1.67 Å) induces a rotation of the Sn–I octahedra, thereby shifting the band gap to a larger value compared to MASnI_3_.^36,40,41^

The PL decay of all compounds extends to several nanoseconds (Figure 2d). While broad PL fwhm’s (>130 meV), high absorption onsets (>1.5 eV) and low carrier lifetimes (tens to hundreds of picoseconds) have been reported for these three compounds,^17,42−45^ our relatively long lifetimes, narrow PL (Table S1), and narrow optical band gaps, suggest a relatively low defect- and doping density^17,18^ and are in good agreement with results in previous works resulting in well-performing solar cells.^24,30,33,46−48^

Figure 2e shows the XRD patterns for all compositions. The slightly lower diffraction angles for FASnI_3_ compared to MASnI_3_ confirm the previously described longer Sn–I bond length in FASnI_3_, leading to a higher octahedral volume and larger lattice spacing. The narrow and intense XRD peaks show that the final films of all three compounds are highly crystalline. Moreover, the very dominant {001} peaks in the hybrid compounds and the {020} and {101} peaks in CsSnI_3_ indicate strong preferential growth of the perovskite with the octahedra oriented parallel with respect to the substrate.

We underline that the results presented in this work suggest a material quality of CsSnI_3_ and MASnI_3_ that are on par with FASnI_3_, allowing for meaningful conclusions by comparing the photophysics of these three materials.

Figure 3a,b displays normalized photoluminescence spectra under various excitation densities for FASnI_3_ and CsSnI_3_ (see Figure S10 for MASnI_3_). In both compounds, the PL emission peak shifts to higher energy and broadens when excited from approximately 0.22 μJ cm^–2^ (∼9 × 10^16^ cm^–3^ carrier density, SI note I, Figure S15) onward, resulting in an increased emission at higher energy. The increased contribution toward higher energy indicates the presence of long-lived hot carriers, i.e., prolonged presence of carriers with excess energy with respect to the band edge, such that they radiatively recombine from their higher-energy state.^7,8,11,18,49^ We note that this effect is dynamic and is not caused by laser-induced doping or disorder in our samples; low-fluence measurements performed on the same spot after high-fluence excitation reveal only minute changes with respect to a “fresh” low-fluence measurement (Figure S9), whereas any degradation effects are expected to lead to a permanent Burstein–Moss effect.^34,35,50^

**Figure 3:**
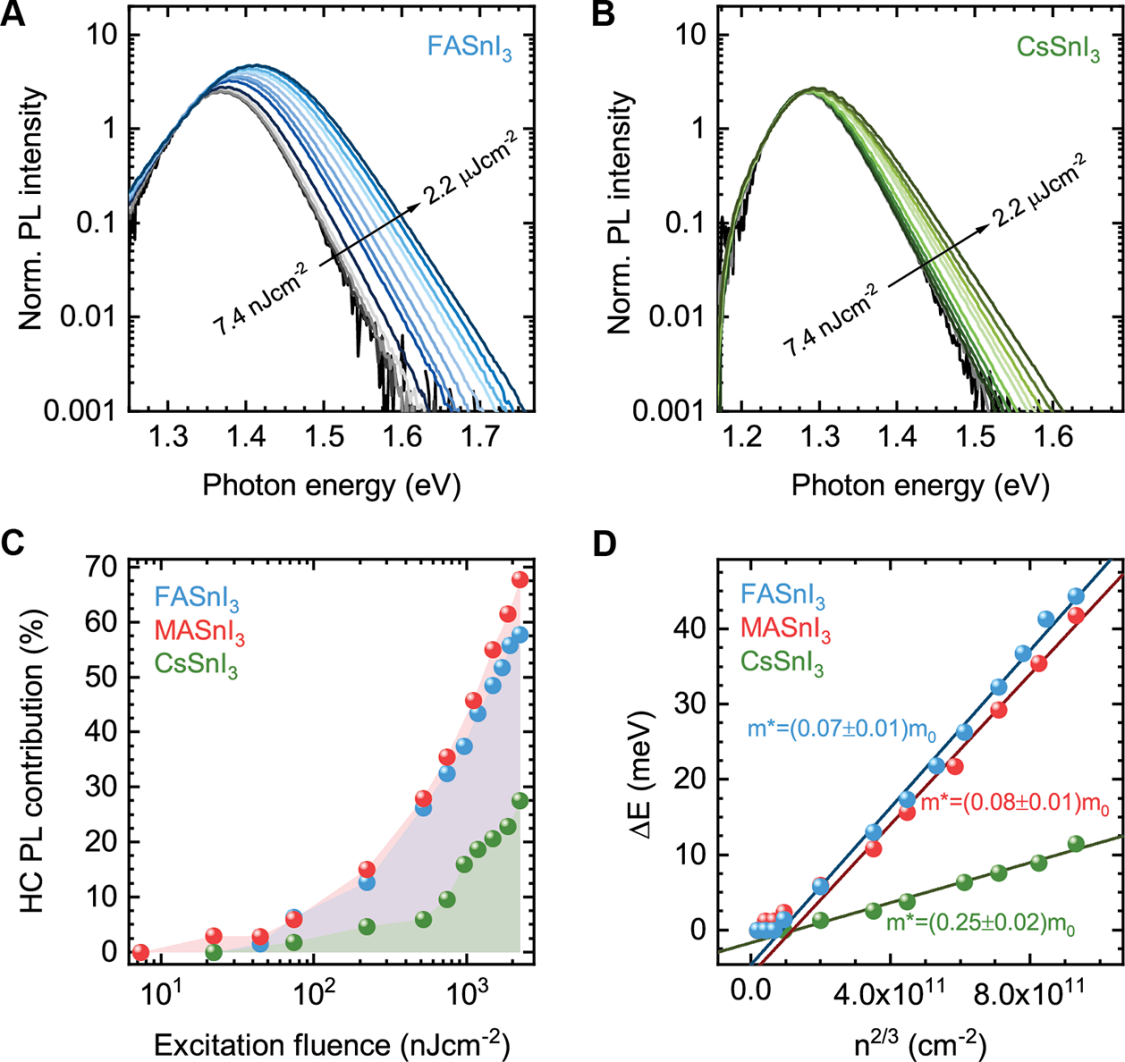
Semilog plot of fluence-dependent photoluminescence spectra of (a) FASnI_3_ and (b) CsSnI_3_. Spectra were normalized at 1.310 eV for FASnI_3_ and at 1.238 eV for CsSnI_3_. (c) The hot-carrier PL contribution as a function of the fluence and (d) extracted blueshift of the emission peak as a function of the photocarrier density *n*^2/3^ for all three compounds.

In Figure 3c we show the contribution of the hot-carrier photoluminescence to the total emission, i.e., the relative increase in PL intensity at the high-energy side when increasing the fluence. The compounds have strikingly different trends, where the hybrid perovskites have approximately double the contribution of the inorganic perovskite, illustrating the importance of the A-site cation on the hot-carrier dynamics. In addition, in Figure 3d we observe that the blueshift extracted from the fluence-dependent PL spectra is linear to the photocarrier density to the two-thirds power (*n*^2/3^) (see Figure S11 for the blueshift as a function of the fluence). This particular relationship has been attributed to a dynamic photon-induced band-filling, also called a dynamic Burstein–Moss effect.^11,51,52^

As the photogenerated carriers with excess energy cool via the emission of LO phonons, band-edge states gradually fill up and saturate. This inhibits other carriers at higher energy from cooling further to the band edge. The time for which the hot-carrier population is maintained depends on the electronic density of states near the band edges, the density of photogenerated carriers, and the overall photogenerated carrier lifetime. If sufficiently long, then the hot carriers residing at higher band energies recombine radiatively, emitting a higher-energy photon. In this model, increasing the photogenerated carrier density would lead to a progressively strong filling of band-edge states, resulting in a shift of the quasi-Fermi level over the band edges and thus in an effective blueshift of the optical band gap. Furthermore, from the slope of the linear fits in Figure 3d we determine the reduced effective masses to be (0.07 ± 0.01)*m*_0_ for FASnI_3_, (0.08 ± 0.01)*m*_0_ for MASnI_3_, and (0.25 ± 0.02)*m*_0_ for CsSnI_3_ (SI note II). These values are in line with reported calculated and experimental values that range between 0.07*m*_0_ and 0.2*m*_0_.^37,38,53−56^

Time-resolved photoluminescence measurements allow us to track the evolution of the hot-carrier emission after initial high-fluence excitation (2.2 μJ cm^–2^, ∼9 × 10^17^ cm^–3^). Figure 4a,b shows energy-dependent photoluminescence spectra normalized at the red tail, taken at different delay times after excitation for CsSnI_3_ and FASnI_3_. We observe that the emission peak shifts to lower energy and the high-energy tail reduces, rendering a more symmetrical PL profile with time. We attribute this to the relaxation of hot carriers.^11,18^

**Figure 4:**
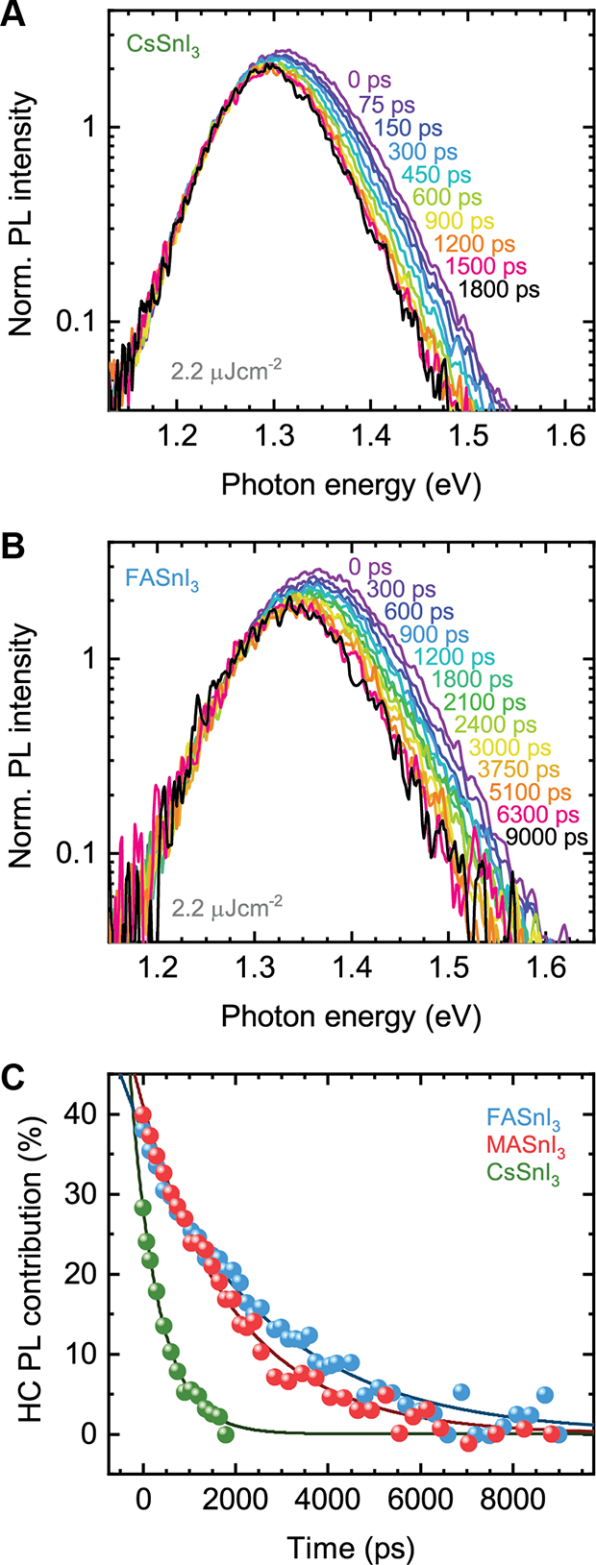
Energy-dependent PL spectra at indicated times after initial 2.2 μJ cm^–2^ laser pulse excitation, normalized at the red tail for (a) CsSnI_3_ and (b) FASnI_3_. (c) The hot-carrier PL contribution to the overall emission as a function of time, for all three compounds.

We note that relaxation of carriers from in-band trap states as an explanation for the observed phenomena is incompatible with the higher hot-carrier PL contribution and slower cooling observed in less defective tin-based perovskites.^18,20^ In addition, the optical transition cross section of the trap state would have to be unusually high to cause the pronounced blueshifts. Lastly, neither a higher-energy resonance nor saturation of the trap state is observed during 24 K carrier-dependent PL measurements.^11^ Carrier diffusion and subsequent self-absorption cannot explain the initial blueshift (and the much more pronounced redshift over time) upon carrier-density increase and play a minor to negligible role (SI note III, Figures S16–S20).

For CsSnI_3_ no further spectral changes are observed after about 1500 ps, suggesting that the hot-carrier cooling extends to 1500 ps. This is further verified by a similar shape of the spectrum late in time (1800 ps) and a low fluence measurement on the same spot (Figure S12a) and by the overlap of the PL decay at different energies across the spectrum at about 1400 ps (Figure S12b). For the latter, a faster decay at the high-energy side indicates vibrational relaxation to lower-lying states, i.e., cooling, as an additional decay channel, whereas the same decay rate across the spectrum indicates solely radiative band-to-band transition.

For FASnI_3_ and MASnI_3_, the spectral evolution took a considerably longer time and, thus, was probed over a 10 ns time range. Figure 4b exhibits that FASnI_3_’s emission profile redshifts on a much longer time range of about 6300 ps, indicating a slower hot-carrier cooling time in comparison to CsSnI_3_. This is again further checked by an overlap of the PL decay curves across the spectrum at about 6000 ps (Figure S13a). By the same analysis, we find that it takes about 4000 ps for the carriers in MASnI_3_ to be fully cooled (Figure S14a).

To track the decay of the hot-carrier PL contribution over time, we define a fully cooled spectrum at the end of our time range as the spectrum with zero hot-carrier PL contribution and extract the relative contribution of the high-energy emission as a function of time relative to that spectrum as a fraction of the total integrated intensity. This is shown in Figure 4c for all three compounds. Here, we observe again that the hybrid perovskites have a stronger hot-carrier PL contribution, and in addition, this contribution decays slower than in CsSnI_3_. Through a single-exponential fit going to zero, a decay constant of τ_1_ = 2.8 ± 0.1 ns, τ_1_ = 2.0 ± 0.1 ns, and τ_1_ = 0.61 ± 0.02 ns is found for FASnI_3_, MASnI_3_, and CsSnI_3_ respectively. Interestingly, these observations of a stronger hot-carrier contribution and a slower cooling in the hybrid perovskites as compared to the fully inorganic one have similarly been found in lead-based perovskites by Zhu et al. and Yang et al.^7,9^

This hot-carrier cooling lifetime is on the order of the radiative recombination lifetime. If dynamic band-filling is the dominant cause for the observed phenomena, this is not surprising; hot carriers can only relax once the carrier density is sufficiently reduced through recombination such that the lower-lying energy states get depleted.^49,57^ However, considering the band-filling effect, the observed difference in the hot-carrier PL contribution between CsSnI_3_ and FASnI_3_, MASnI_3_ is of unclear origin; it has been shown that the A-site cation only indirectly, through imposed distortions on the octahedra or octahedral volume changes, influences the electronic structure, and thus, a large variation in the electronic density of states near the band edge is not expected.^58^

The cation influence on hot-carrier cooling in lead-based perovskites has been linked to differences in the phonon band structure of the hybrid and inorganic perovskite, e.g., the presence of an overlap of a particular LO phonon mode of the organic cation with acoustic phonon modes facilitating phonon reabsorption to reheat carriers or differences in phonon density of states resulting in variations of the phonon decay pathways.^9,13^ Additionally, a difference in the defect density could lead to additional relaxation pathways for the carriers and a reduced filling of the energy states in the case of the inorganic compound. Given our described successful efforts in obtaining high-quality films for all three perovskites, we believe that the difference in behavior should be attributed to the phononic properties of the different A-site cations, but future research should clarify this further.

In conclusion, we report for the first time that exceptional slow cooling of a few nanoseconds is not limited to FASnI_3_; other tin triiodide perovskites exhibit the phenomenon as well. This is achieved through the substantial improvements that were made in the MASnI_3_ and CsSnI_3_ film quality, such that all final ASnI_3_ films exhibit long carrier lifetimes, narrow PL fwhm values, and high crystalline order. Within this film optimization, in particular MASnI_3_ shows a similarly pronounced carrier-density-dependent hot-carrier emission that decays with τ_1_ ≈ 2 ns as compared to τ_1_ ≈ 2.8 ns for FASnI_3_. The hot-carrier emission in CsSnI_3_, on the other hand, decays faster with τ_1_ ≈ 0.6 ns. These results suggest a role for the organic cation in the hot-carrier cooling of tin perovskites.

## Supporting Information Available

Experimental details, including sample preparation, characterization settings, XRD patterns, SEM images, AFM images, and additional results of steady-state and time-resolved PL (PDF)
