# Supplementary material for: Cation Influence on Hot-Carrier Relaxation in Tin Triiodide Perovskite Thin Films
Source: ACS Energy Lett. 2024 Feb 15;9(3):992–9. doi: 10.1021/acsenergylett.4c00055 (PMC10928715; doi:10.1021/acsenergylett.4c00055)
Supplement: Supplementary file 1 — nz4c00055_si_001.pdf [file nz4c00055_si_001.pdf]

Supporting Information Cation influence on hot-carrier relaxation in tin triiodide perovskite thin films

Larissa J.M. van de Ven, Eelco K. Tekelenburg, Matteo Pitaro, Jacopo Pinna, and Maria A. Loi*

Zernike Institute for Advanced Materials, University of Groningen, Nijenborgh 4, 9747 AG, Groningen, The Netherlands

E-mail: m.a.loi@rug.nl

Thin film preparation

3 cm by 3 cm glass substrates were cleaned with a 120 ℃ heated 1:9 soap:deionized water mixture. Subsequently, these substrates were sonicated twice for 10 minutes in DI water, twice for 10 minutes in acetone, and twice for 10 minutes in isopropanol. Substrates were then blown dry using a nitrogen gun and were further dried in a 140 ℃ oven for 15 minutes. The substrates underwent a 20-minute UV-Ozone treatment after which the solutions were deposited on the films within 30 minutes.

Preparation of perovskite precursor solutions and the fabrication of thin films were done in a nitrogen-filled glovebox (H_2O, $O_2 < 0.1$ ppm). The solutions were kept stirring at room temperature until fully dissolved. Solutions were filtered using a 0.2 μm polytetrafluoroethylene (PTFE) filter. All films were spin-coated with 4000 rpm speed, 2000 rpm acceleration, and 60 seconds duration. 100 μl of perovskite solution is deposited on the center of the glass substrates before revolving.

The chemicals used were SnI_2 (Sigma-Aldrich, 99.99%), FAI (TCI, >98%), MAI (TCI, >98%), CsI (Sigma-Aldrich, 99.99%), PEAI (TCI, >98%), SnF_2 (Sigma-Aldrich, 99% and Acros Organics, 99%), anhydrous DEE (TCI, >99.5%), anhydrous Toluene (Acros Organics, >99.8%), anhydrous DMSO (Alfa Aesar, 99.8%), anhydrous DMF (Acros Organics, 99.8%).

Recipe $FASnI_3$

1 M solutions were made by dissolving 1 M FAI, 1 M SnI_2 and 0.1 M SnF_2 (Acros Organics) precursors in mixed solvents of DMSO and DMF (1:4 volume ratio). 500 μl DEE was dropped at the 15th second. Films were annealed at 70 ℃ for 20 minutes.

Recipe 2D/3D $FASnI_3$

1 M solutions were made by dissolving 0.92 M FAI, 0.08 M PEAI, 1 M SnI_2 and 0.1 M SnF_2 (Acros Organics) precursors in mixed solvents of DMSO and DMF (1:4 volume ratio). 500 μl DEE was dropped at the 15th second. Films were annealed at 70 ℃ for 20 minutes.

Starting recipe $MASnI_3$

1 M solutions were made by dissolving 1 M MAI, 1 M SnI_2 and 0.1 M SnF_2 (Sigma-Aldrich) precursors in mixed solvents of DMSO and DMF (1:4 volume ratio). 500 μl DEE was dropped at the 15th second. Films were annealed at 70 ℃ for 20 minutes.

Improved recipe $MASnI_3$

0.7 M solutions were made by dissolving 0.7 M MAI, 0.7 M SnI_2 and 0.07 M SnF_2 (Acros Organics) precursors in mixed solvents of DMSO and DMF (1:4 volume ratio). 650 μl toluene was dropped at the 22nd second. Films were annealed at 70 ℃ for 20 minutes.

Recipe 2D/3D $MASnI_3$

0.7 M solutions were made by dissolving 0.658 M MAI, 0.042 M PEAI, 0.7 M SnI_2 and 0.07 M SnF_2 (Acros Organics) precursors in mixed solvents of DMSO and DMF (1:4 volume ratio). 650 μl toluene was dropped at the 22nd second. Films were annealed at 70 ℃ for 20 minutes.

Starting recipe $CsSnI_3$

1 M solutions were made by dissolving 1 M CsI, 1 M SnI_2 and 0.1 M SnF_2 (Sigma-Aldrich) precursors in mixed solvents of DMSO and DMF (1:4 volume ratio). 500 μl DEE was dropped at the 15th second. Films were annealed at 70 ℃ for 20 minutes.

Improved recipe $CsSnI_3$

1.3 M solutions were made by dissolving 1.3 M CsI, 1.3 M SnI_2 and 0.13 M SnF_2 (Acros Organics) precursors in mixed solvents of DMSO and DMF (1:4 volume ratio). 350 μl toluene was dropped at the 22nd second. Films were put for 10 minutes in the vacuum chamber, followed by 10 minutes annealing at 70 ℃.

Recipe 2D/3D $CsSnI_3$

1.3 M solutions were made by dissolving 1.235 M CsI, 0.065 M PEAI, 1.3 M SnI_2 and 0.13 M SnF_2 (Acros Organics) precursors in mixed solvents of DMSO and DMF (1:4 volume ratio). 350 μl toluene was dropped at the 22nd second. Films were put for 10 minutes in the vacuum chamber, followed by 10 minutes annealing at 70 ℃.

Films were stored between fabrication and measurements in a nitrogen-filled "dry" glovebox (H_2O, $O_2 < 0.6$ ppm).

Photoluminescence spectroscopy

A 532 nm laser (second harmonic of Nd:YAG) was used to pump a mode-locked Ti:sapphire laser (Mira 900, Coherent). The Ti:sapphire laser generates ultrashort laser pulses (Δt < 150 fs) of 800 nm with a repetition rate of 76 MHz. A second harmonic generator was used to double the photons' energy to 3.1 eV. Using an iris of 2 mm the excitation beam was spatially limited. Using a neutral density filter wheel the excitation fluence was adjusted and the power was measured directly thereafter. A lens was used to focus the beam on the samples and measurements were carried out under inert atmosphere. The samples were measured in reflection geometry. The photoluminescence is collected and focused into the detector using achromatic doublets. A spectrometer with a 30-50 lmm^{-1} grating was used to disperse the photoluminescence. The steady-state spectra were recorded with a spectrally-calibrated CCD camera (Hamamatsu) and the time-resolved spectra were recorded with a NIR sensitive streak camera (Hamamatsu). For carrier lifetime determination throughout the optimization of our films, single-sweep mode is used in combination with a pulse picker to reduce the repetition rate. Time-resolved energy-dependent spectra are not spectrally calibrated. The time resolution of the 2 ns time range of Synchronscan mode is about 20 ps and for the 10 ns range of single sweep mode is around 1% of the time window, i.e. about 100 ps. In the 2 ns time range, the energy-resolved spectra taken at different times after excitation

(as indicated in Figure 4 of the main text) are extracted using horizontal, energy-resolved cuts with an ROI of 8 ps. For the 10 ns time range the ROI used was 60 ps.

UV-VIS absorbance spectroscopy

Absorption spectra of the perovskite films are measured with a Shimadzu UV-3600 UV-Vis-NIR spectrometer in absorbance mode. A tungsten lamp was used as a light source.

X-ray powder diffraction

Powder X-ray diffraction (PXRD) patterns of the thin films were recorded on a Bruker D8 Advance X-ray diffractometer. Cu Kα X-rays with $\lambda = 1.54$ Å were used as the X-ray source. The sample is rotated at 60 rpm during measuring. The geometry of the set-up used is the Bragg Bretano geometry and a Lynxeye detector is used.

Scanning electron microscopy

Scanning electron microscopy (SEM) images were recorded in vacuum on an FEI Helios G4 CX. The accelerating voltage used was 10 kV and 0.69 nA current was used. To reduce charging, silver paste is put on the bottom, side and a bit on the top of the sample to electrically ground the sample.

Atomic force microscopy

Atomic force microscopy (AFM) images were taken with a Bruker multimode eight microscope in ScanAsyst Peak Force Tapping mode. The probes used were ScanAsyst air probes (resonant frequency 70 kHz, spring constant 0.4 Nm^{-1}). Micrographs were recorded using a scan rate of 0.988 Hz with a resolution of 512 samples per line. The raw images were analyzed with Gwyddion data analysis software. Image enhancement tools used were correction for horizontal scars, aligning rows, and removing polynomial background (of degree 1).

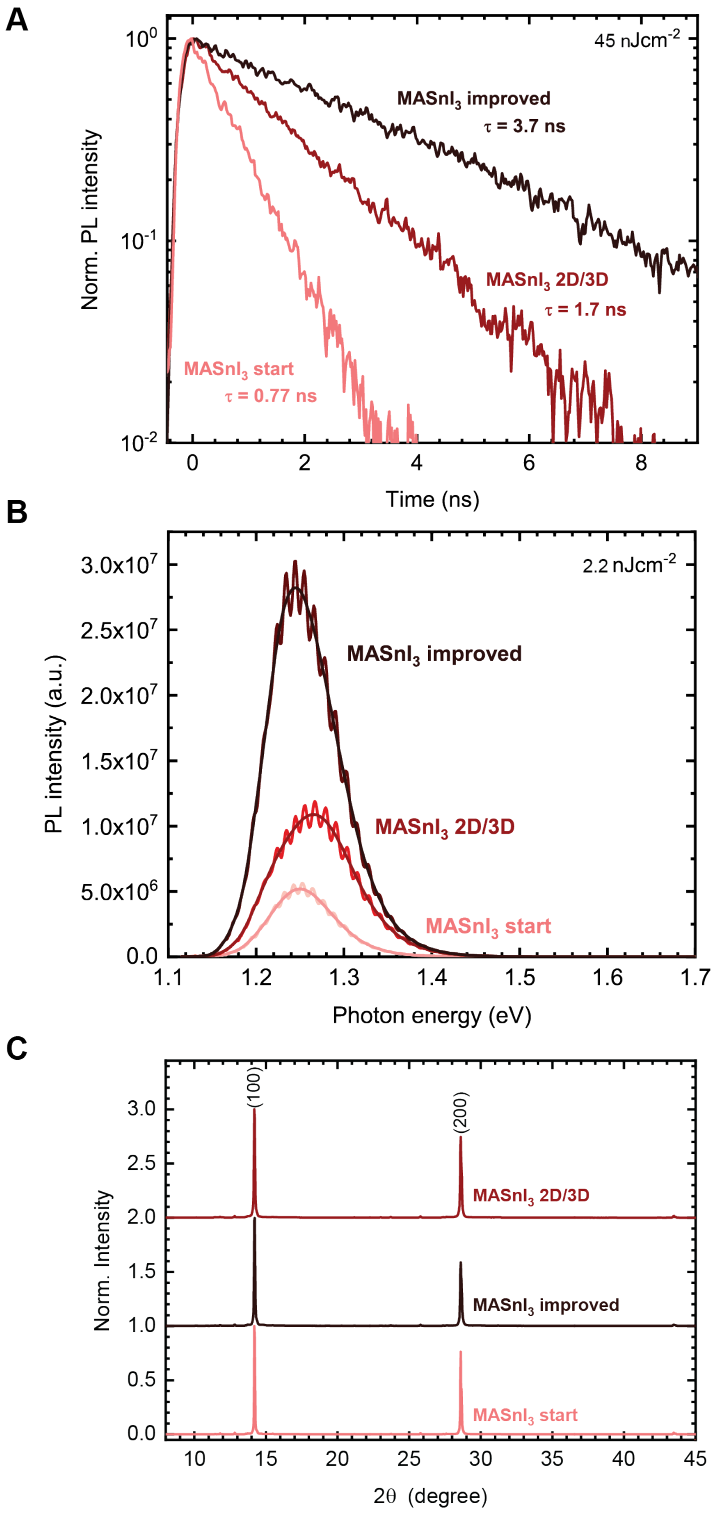

Supplementary Figure 1: (a) Steady-state and (b) time-resolved PL over the course of the improvements in $MASnI_3$, i.e. going from start recipe to improved recipe to the 2D/3D recipe. An interference pattern in the steady-state PL is observed (lighter shade) and its smoothened curve (9 pts FFT filter) is displayed in darker shade. (c) XRD pattern for all $MASnI_3$ versions, peaks are assigned according to the $Pm\overline{3}m$ cubic structure.[1]

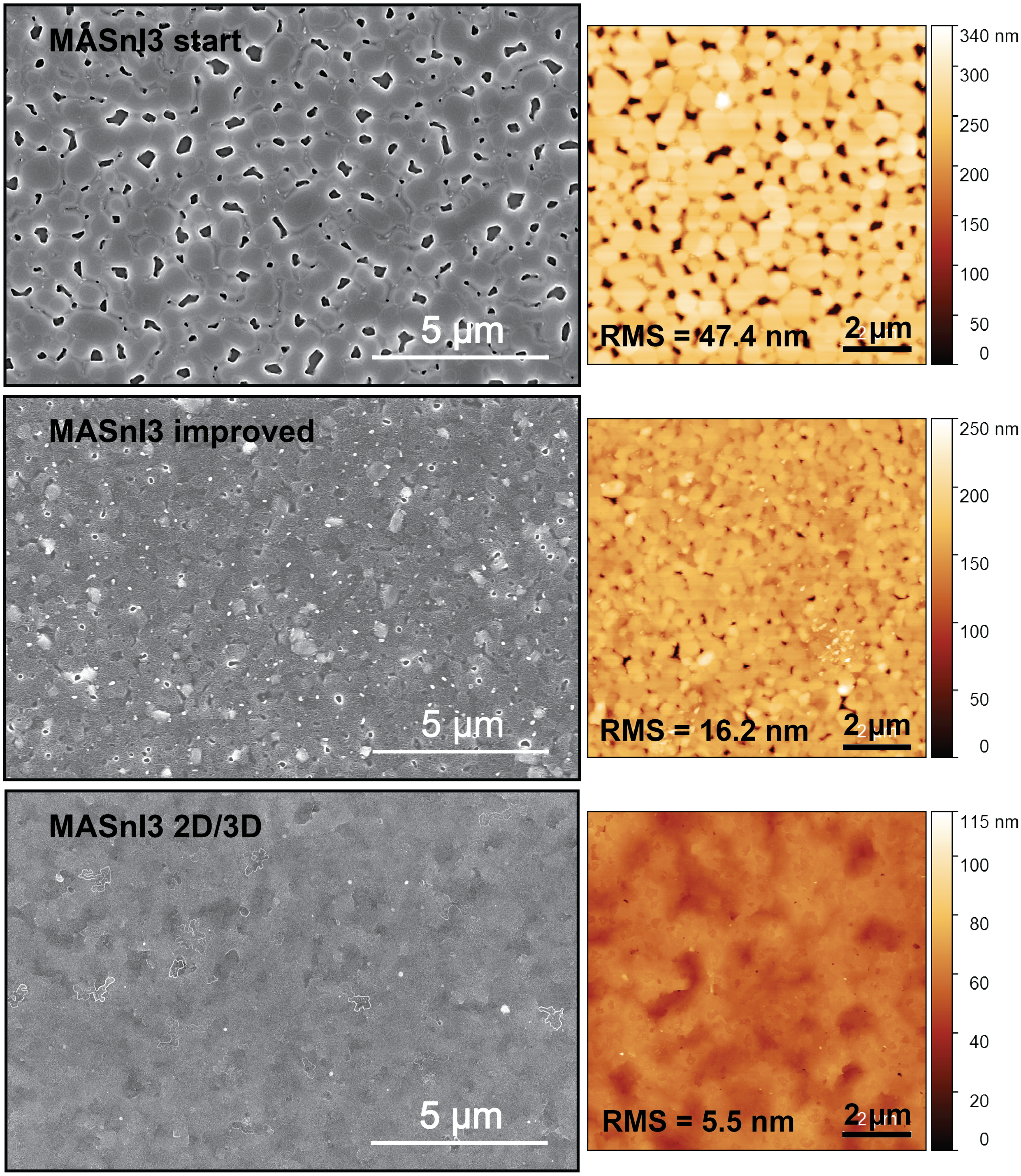

Supplementary Figure 2: SEM (left column) and AFM (right column) micrographs of $MASnI_3$ prepared with starting recipe (top row), improved recipe (middle row), and with 6 mol% PEAI substitution (2D/3D) (bottom row).

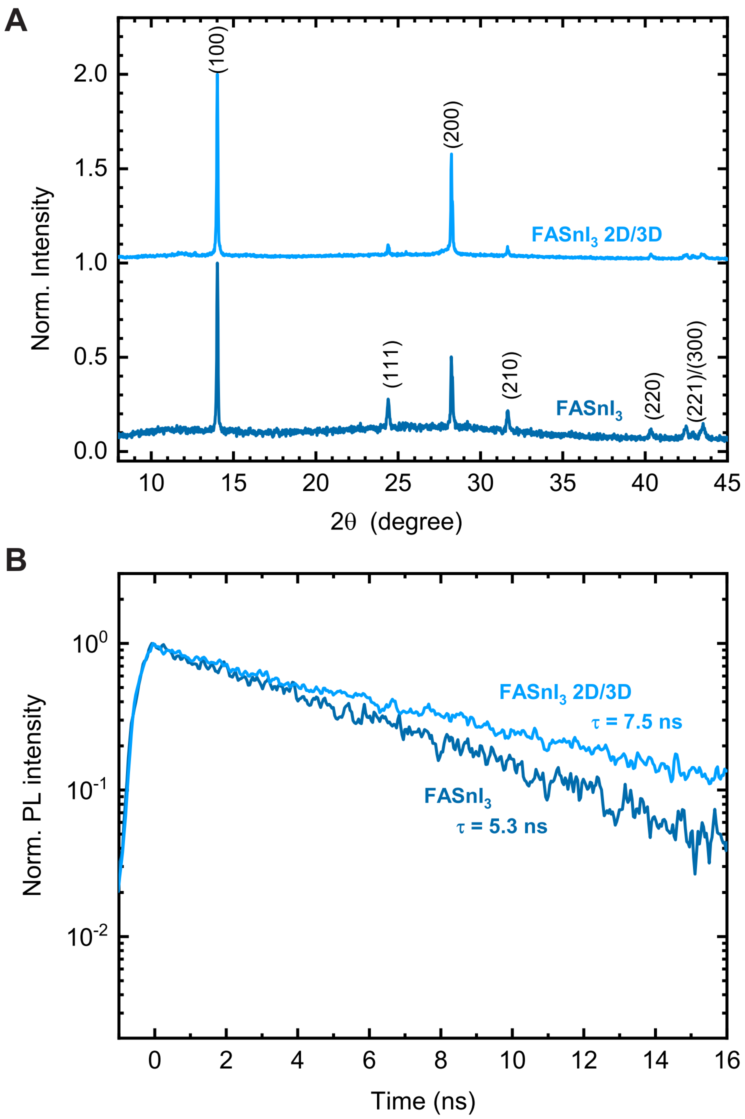

Supplementary Figure 3: (a) XRD patterns for $FASnI_3$ and $FASnI_3$ with 8 mol% PEAI substitution (2D/3D), and (b) time-resolved PL spectra for both, taken with $25\,\mathrm{nJcm^{-2}}$. XRD peaks are assigned according to the $Pm\overline{3}m$ cubic structure of $FASnI_3$.[2]

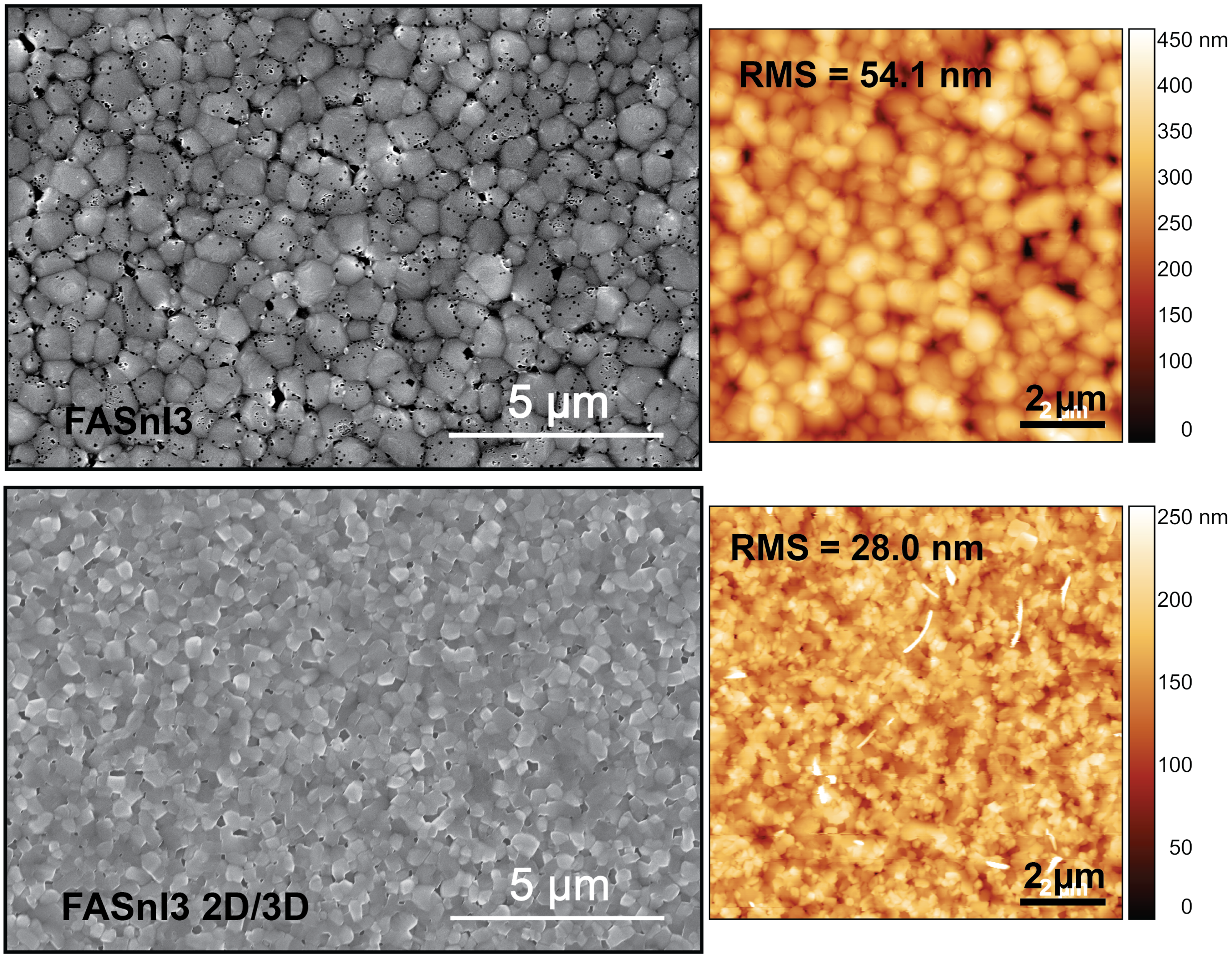

Supplementary Figure 4: SEM (left column) and AFM (right column) micrographs of $FASnI_3$ (top row) (small holes within the grain are expected to be degradation effects while performing the experiment) and with 8 mol% PEAI substitution (2D/3D) (bottom row).

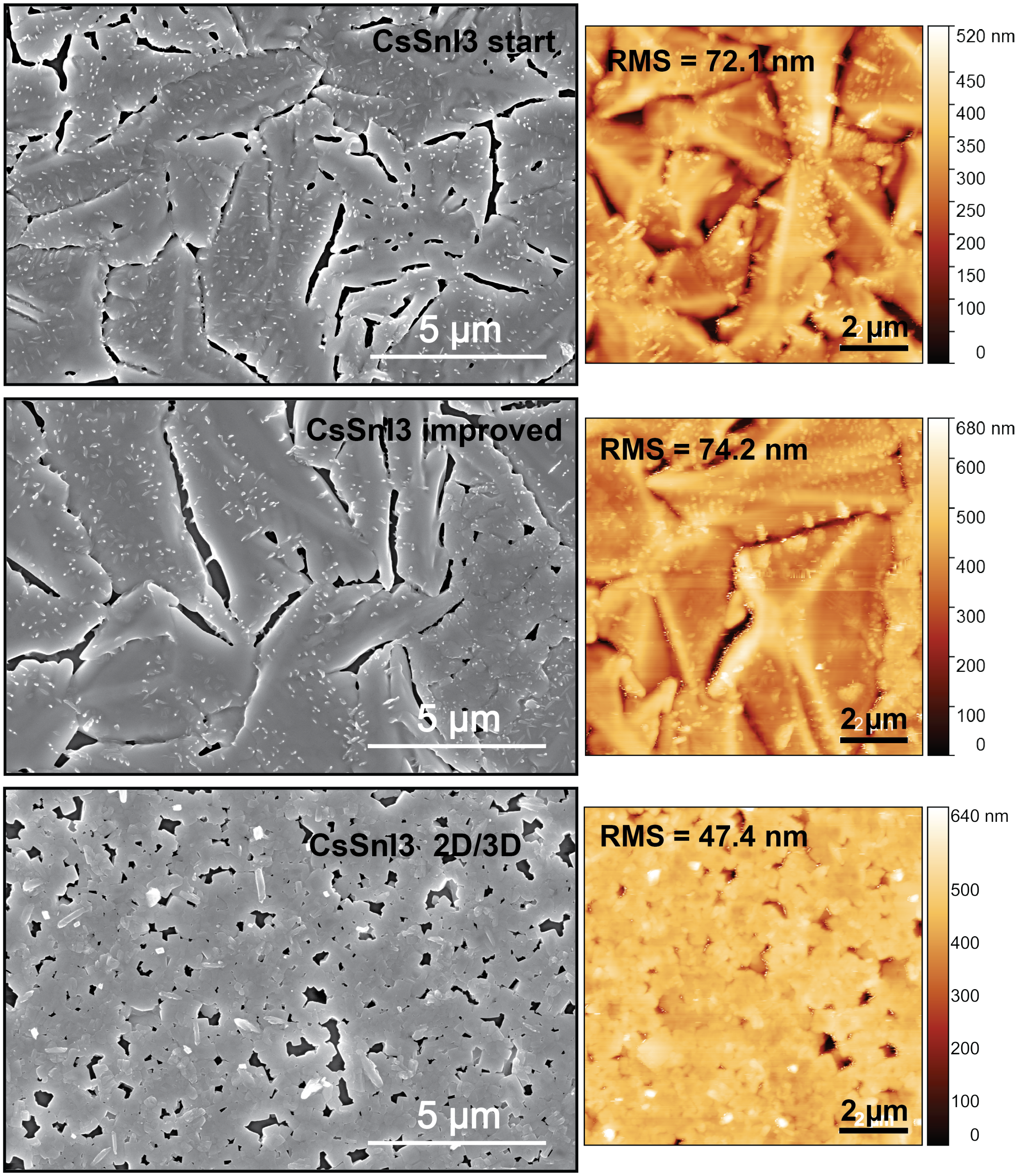

Supplementary Figure 5: SEM (left column) and AFM (right column) micrographs of $CsSnI_3$ prepared with starting recipe (top row), improved recipe (middle row), and with 5 mol% PEAI substitution (2D/3D) (bottom row). 2D/3D and improved 3D showed fluctuations in their morphology but the 2D/3D method provided better reproducibility of the thin films.

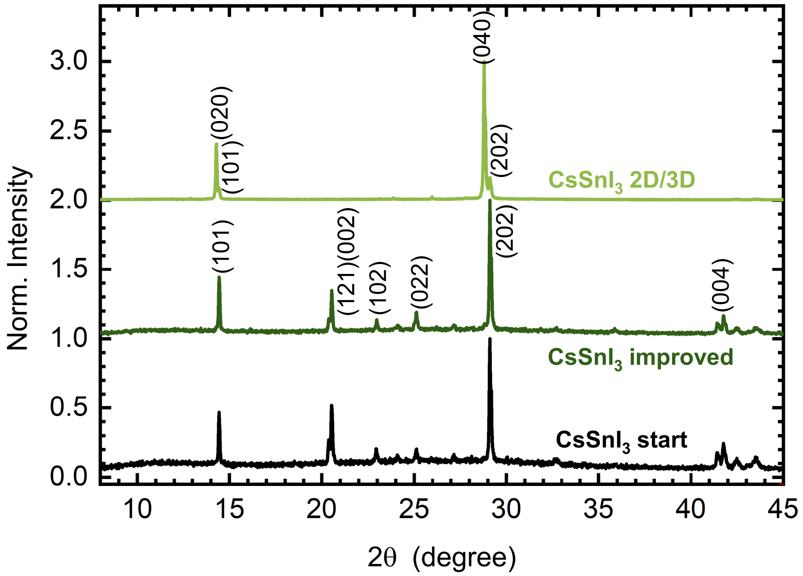

Supplementary Figure 6: XRD patterns for $CsSnI_3$ prepared with starting recipe, improved recipe, and with 5 mol% PEAI substitution (2D/3D). Peaks are assigned according to the Pnma orthorhombic structure of $CsSnI_3$.[3]

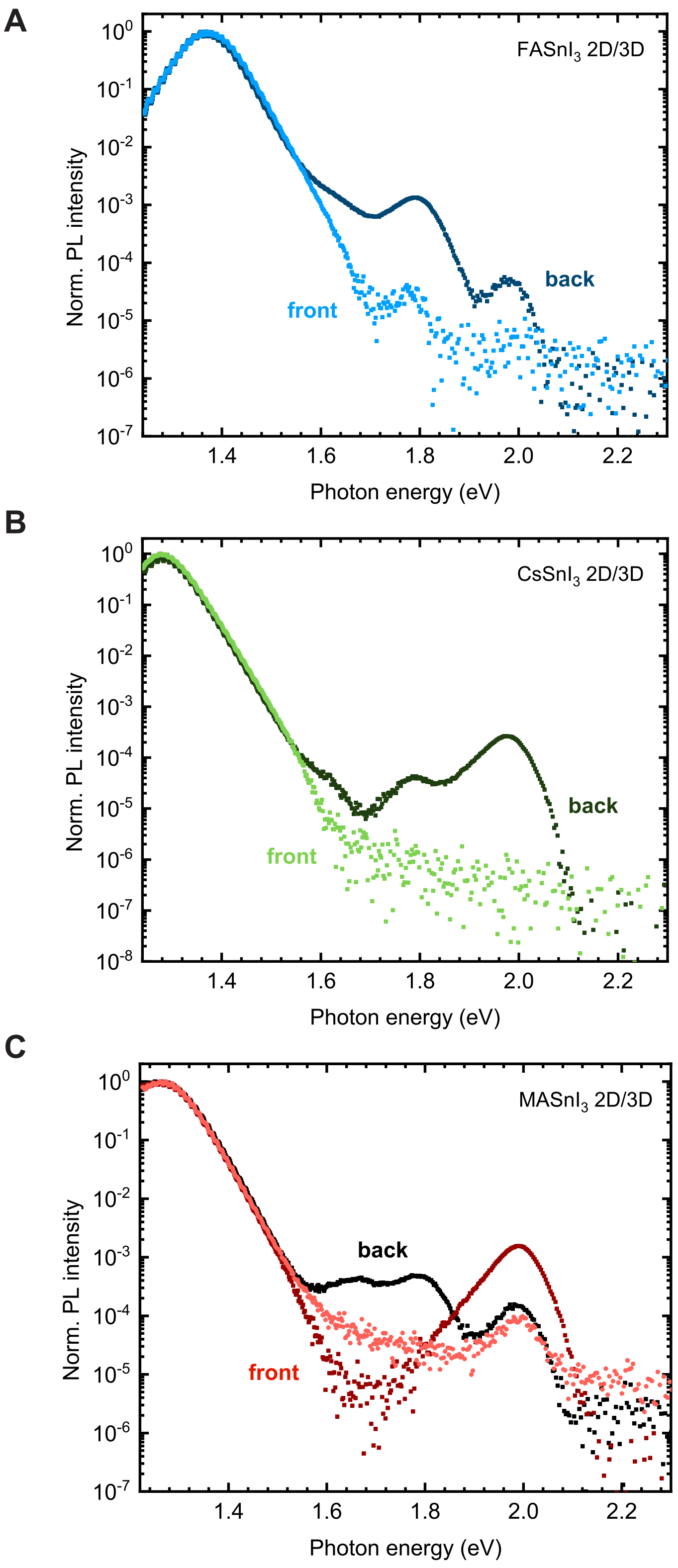

Supplementary Figure 7: Steady-state PL measurements both on the front (air/perovskite interface) and back (perovskite/glass interface) of 2D/3D $FASnI_3$ (a), 2D/3D $CsSnI_3$ (b), and 2D/3D $MASnI_3$ (c). For $MASnI_3$ front measurements on two different film spots are shown.

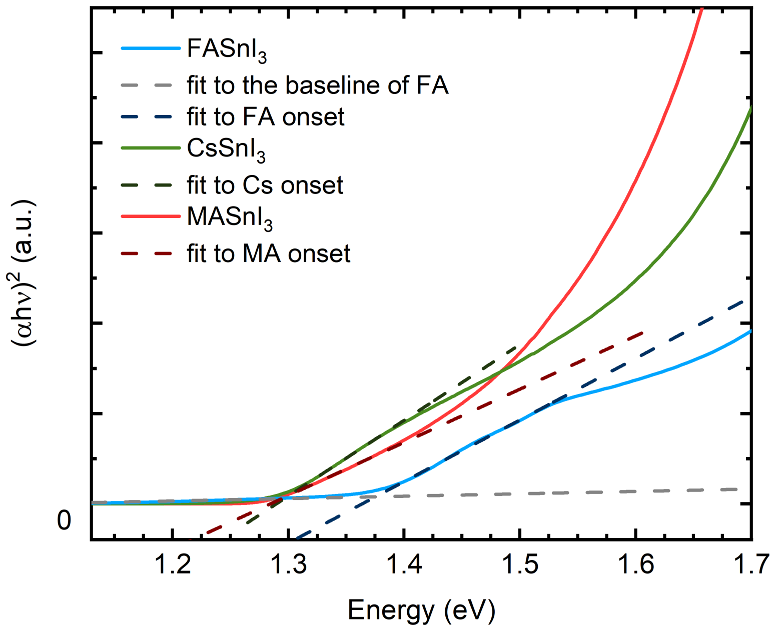

Supplementary Figure 8: Tauc plot for direct bandgap semiconductors of $FASnI_3$, $CsSnI_3$, and $MASnI_3$. The band gap of $FASnI_3$ is determined by the baseline approach by Makula et al.[4] Band gaps are estimated to be 1.38 eV, 1.29 eV, and 1.28 eV for $FASnI_3$, $CsSnI_3$, and $MASnI_3$ respectively.

Supplementary Table 1: Photoluminescence peak position and full-width half-maximum for the three compounds: $FASnI_3$, $CsSnI_3$, and $MASnI_3$.

	PL peak (eV)	PL FWHM (meV)	E_g (eV, from Tauc)
$FASnI_3$	1.37	104	1.38
$CsSnI_3$	1.28	84	1.29
$MASnI_3$	1.25	88	1.28

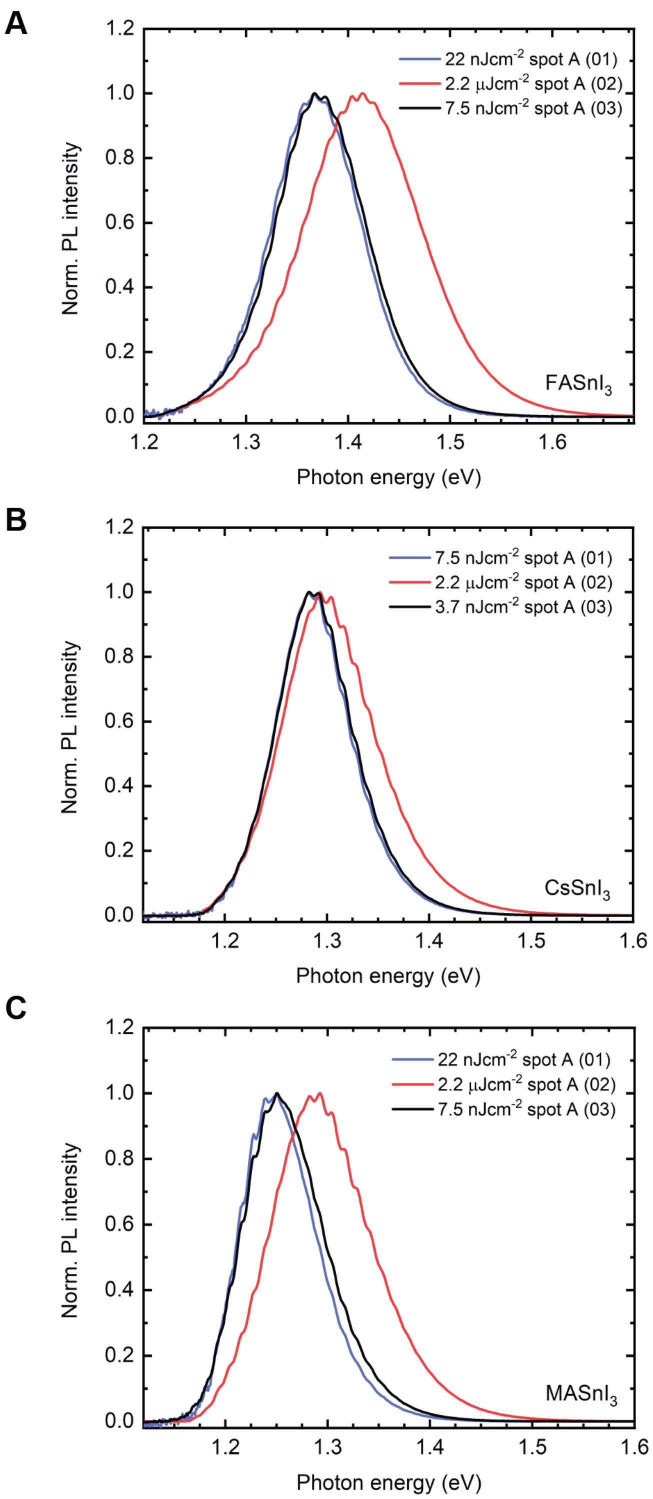

Supplementary Figure 9: Low fluence measurements performed before and after a high fluence ($2.2\,\mu$Jcm^{-2}) measurement, on the same spot for $FASnI_3$ (a), $CsSnI_3$ (b), $MASnI_3$ (c). Performed to check for any laser-induced degradation effects on the shape of the spectra.

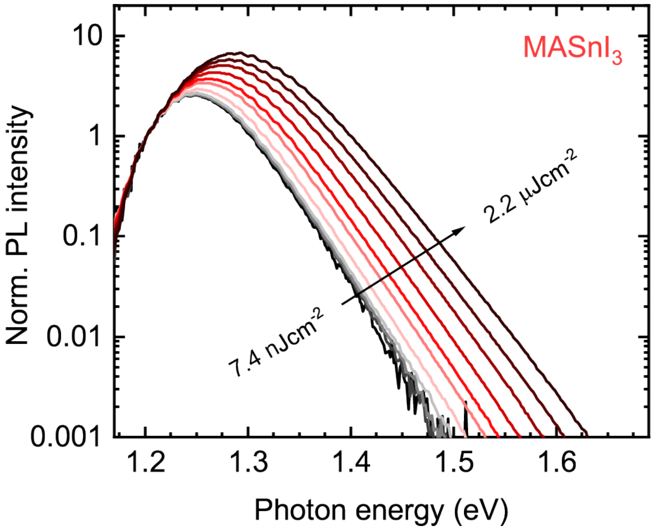

Supplementary Figure 10: Semi-log plot of fluence-dependent photoluminescence spectra of $MASnI_3$. Fluence was varied from 7.4 nJcm^{-2} to 2.2 μJcm^{-2}. Spectra were normalized at 1.202 eV.

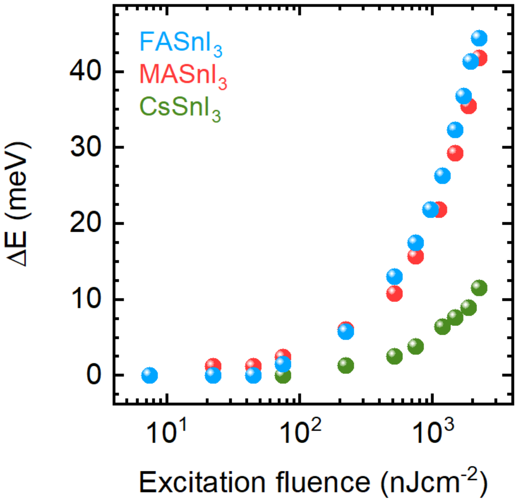

Supplementary Figure 11: Blueshift of the emission peak as a function of the laser fluence, for all three compounds.

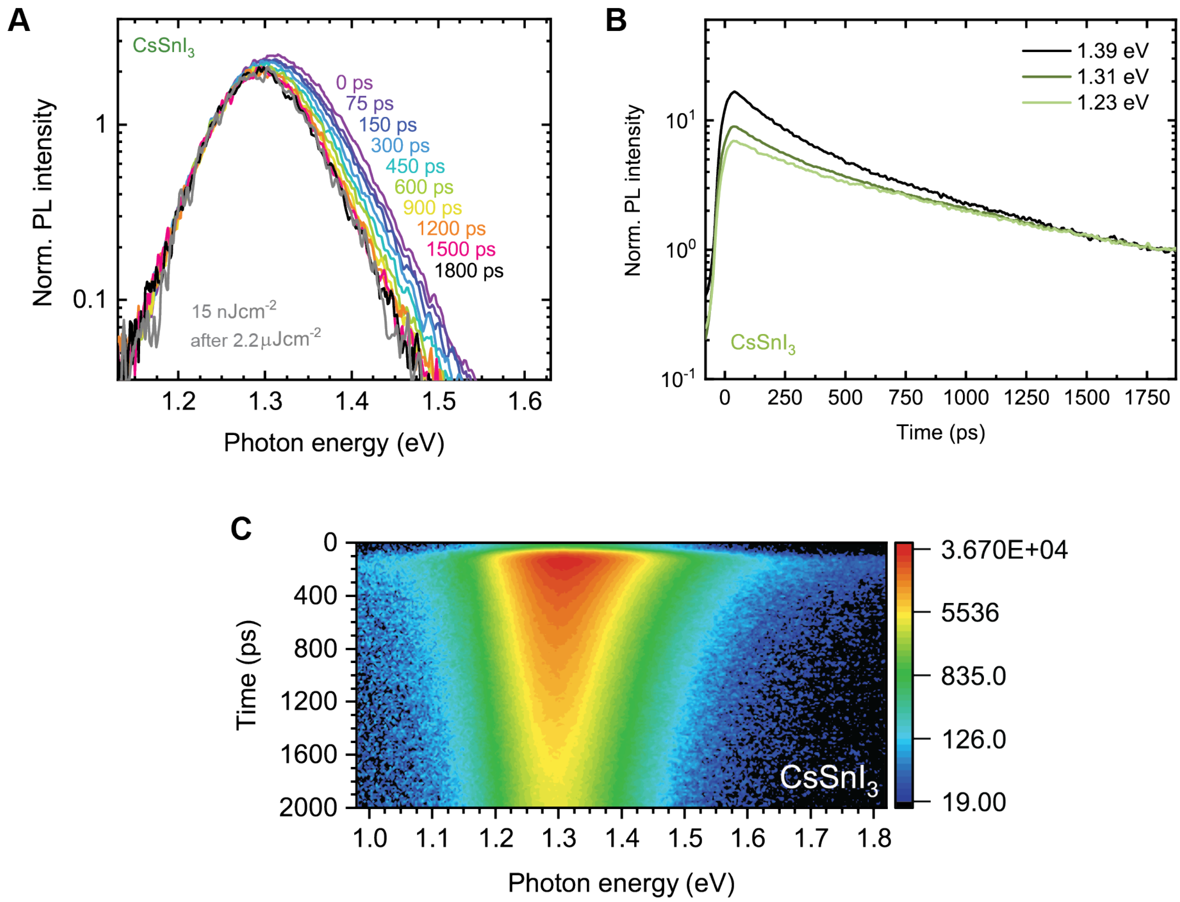

Supplementary Figure 12: Overview of time-resolved data of $CsSnI_3$ at high fluence $2.2\,\mu Jcm^{-2}$. (a) Energy-resolved PL spectra taken at indicated times after initial laser excitation. Temporal width of the taken energy-resolved spectra taken is 8 ps. Spectra are normalized at 1.240 eV to show the evolution of the hot-carrier PL clearly. The grey-colored spectrum is an additional, different low-fluence measurement performed on the same spot after high-fluence excitation. (b) PL intensity decays taken at different, specified energies, i.e. at the peak and at the low and high energy side of the spectrum. Spectra are normalized to the tail and the energy width of the decay is 30 meV. (c) False-color (logarithm of intensity) plot of the $2.2\,\mu Jcm^{-2}$ fluence TR-PL spectrum, from which the energy- and time-resolved spectra are taken.

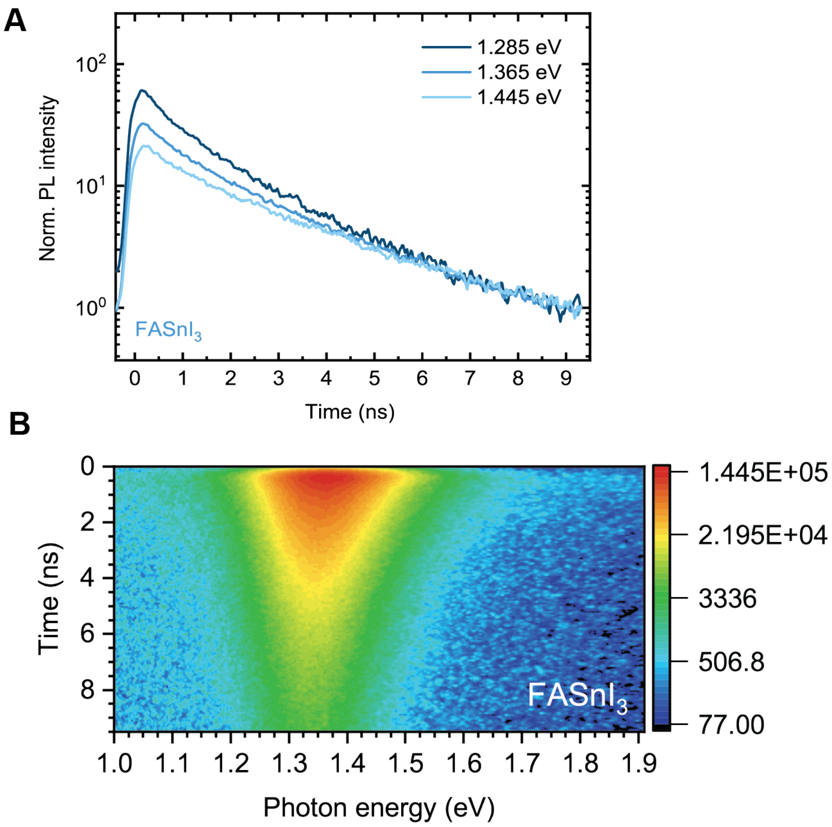

Supplementary Figure 13: Overview of time-resolved data of $FASnI_3$ at high fluence $2.2\,\mu Jcm^{-2}$. (a) PL intensity decays taken at different, specified energies, i.e. at the peak and at the low and high energy side of the spectrum. Spectra are normalized to the tail and the energy width of the decay is 30 meV. (b) False-color (logarithm of intensity) plot of the $2.2\,\mu Jcm^{-2}$ fluence TR-PL spectrum, from which the energy- and time-resolved spectra are taken.

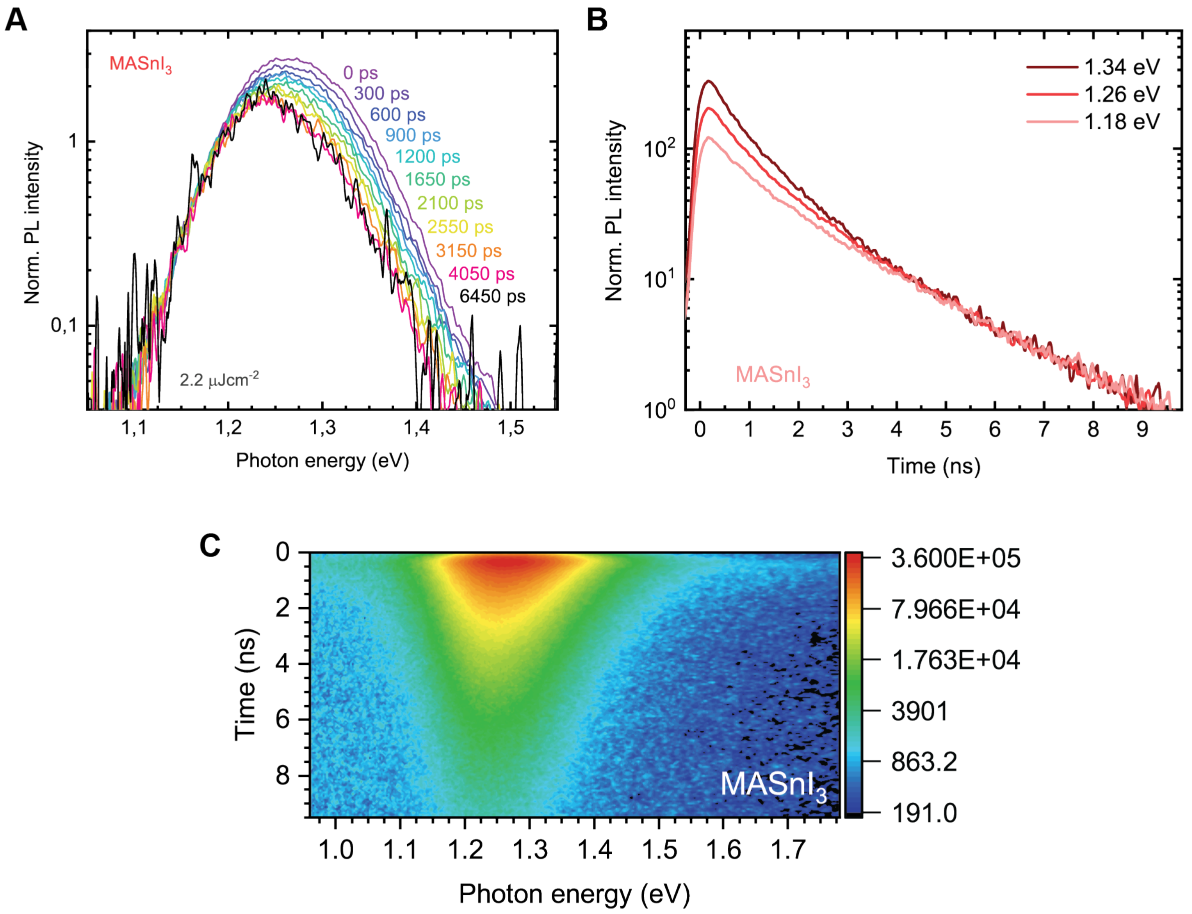

Supplementary Figure 14: Overview of time-resolved data of $MASnI_3$ at high fluence $2.2\,\mu Jcm^{-2}$. (a) Energy-resolved PL spectra taken at indicated times after initial laser excitation. Temporal width of the taken energy-resolved spectra taken is 60 ps. Spectra are normalized at 1.186 eV to show the evolution of the hot-carrier PL clearly. (b) PL intensity decays taken at different, specified energies, i.e. at the peak and at the low and high energy side of the spectrum. (c) Spectra are normalized to the tail and the energy width of the decay is 30 meV. False-color (logarithm of intensity) plot of the $2.2\,\mu Jcm^{-2}$ fluence TR-PL spectrum, from which the energy- and time-resolved spectra are taken.

Supplementary Note I

Fluence and carrier density estimation

With a beam area of $0.018\,\text{mm}^2$, the fluence or excitation energy density is calculated by:

$$\text{fluence} = \frac{P_{\text{avg}} \cdot (1/f_{\text{rep}})}{0.00018\,\text{cm}^2},$$

where P_{avg} is the excitation power and f_{rep} the repetition rate of the laser.

The carrier density is then estimated by:

$$\text{carrier density} = \frac{P_{\text{avg}} \cdot (1/f_{\text{rep}}) \cdot \alpha}{E_{\text{ph}} \cdot 0.00018\,\text{cm}^2},$$

where α is the absorption coefficient at the excitation energy and E_{ph} the excitation photon energy. In literature, a broad range of absorption coefficient values for tin perovskites is reported: $\sim 3.3 \times 10^4$ to $\sim 2.6 \times 10^5$.[5–8] To get an indication of which end of the range applies to the materials used in this research, UV/VIS spectroscopy measurements were performed.

The absorption coefficient can be defined from Beer-Lambert's exponential law:

$$\frac{I_t}{I_0} = e^{-\alpha t},$$

$$\alpha = \frac{\ln(\frac{I_0}{I_t})}{t}.$$

where t is the film thickness and I_0, I_t is the incoming and transmitted intensity. The absorbance (A) obtained from our UV/VIS spectroscopy measurement is decadic (10-based, i.e. $\log(I_0/I_t)$). This can be transformed to Napierian through:

$$\ln(\frac{I_0}{I_t}) = \ln(10) \cdot \log(\frac{I_0}{I_t}) = \ln(10) \cdot A$$

and thus:

$$\alpha = \frac{\ln(10) \cdot A}{t}$$

Figure 15 shows the absorption coefficient as a function of wavelength for all three compounds. Below approximately 550 nm the detector saturates, meaning that we are experimentally limited. We thus note that we can only obtain a crude indication of the absorption coefficient (and thus carrier density) at our excitation wavelength (400 nm). Considering that it is in line with reports on the higher end of the above-described literature range, we assumed the absorption coefficient to be equal for all three compounds and equal to $2 \times 10^5\,\mathrm{cm}^{-1}$.[6–8] The error in Figure 3d of the main text encompasses, together with the fit error, an error of $0.5 \times 10^5\,\mathrm{cm}^{-1}$ in the absorption coefficient determination.

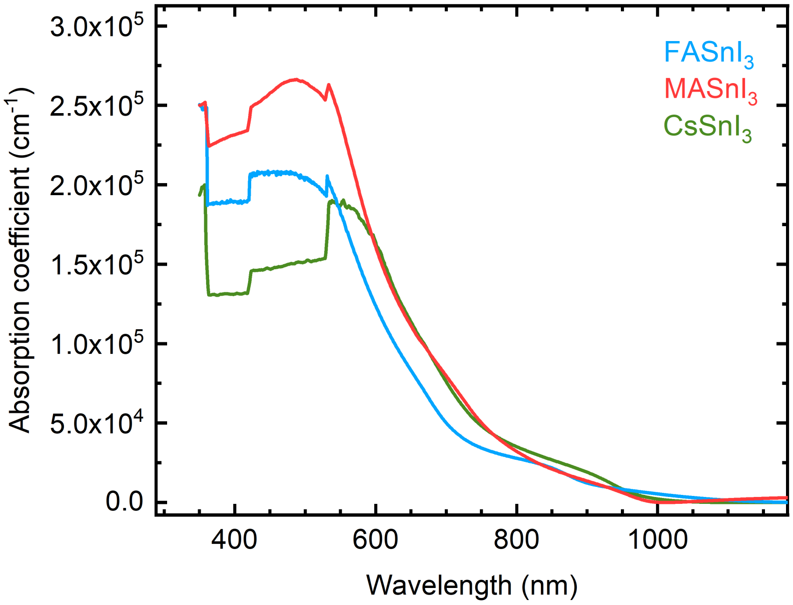

Supplementary Figure 15: Absorption coefficient as a function of wavelength for all three compounds: $FASnI_3$, $MASnI_3$, and $CsSnI_3$. Below approximately 550 nm detector saturation occurs.

In addition, we would like to note here that in previous work of our group on long-lived hot-carrier emission in $FASnI_3$ the beam area was estimated by assuming an ideal Gaussian beam and perfect focus.[9] This leads to a much lower beam area of $0.0011\,\mathrm{mm}^2$ leading to much higher fluence and carrier density than reported here. For the same average laser power and using the same exact measurement set-up, our results on the PL blueshift of $FASnI_3$ are in excellent agreement with what was previously reported.[9]

Supplementary Note II

Optical band gap shift in the band-filling effect

In the band-filling effect, increasing the photogenerated carrier density would lead to a progressively strong filling of band-edge states, resulting in a progressively strong shift of the quasi-Fermi level over the band edges and thus in an effective blueshift of the optical band gap. From parabolic-band theory this carrier-induced Burstein-Moss shift of the optical band gap (ΔE_g^{BM}) can be modeled according to the following equation:[10]

$$\Delta E_g^{BM} = \frac{\hbar^2}{2m_{eh}^*}(3\pi^2 n)^{\frac{2}{3}}, \tag{1}$$

where $\hbar$ is the reduced Planck constant, m_{eh}^* the reduced effective mass (i.e. $\frac{1}{m_{eh}^*} = \frac{1}{m_h^*} + \frac{1}{m_e^*}$) and n the photocarrier density. Thus, by a linear fit of the shift ΔE_g^{BM} vs. $n^{2/3}$, m_{eh}^* can be determined from the slope. Herein we assume a similar effective hole (m_h^*) and electron mass (m_e^*) for $ASnI_3$ such that both carriers contribute to the carrier-induced band-filling effect.

Supplementary Note III

Diffusion and self-absorption

Directly following excitation by a laser pulse most of the carriers will be generated at the top of the film due to the strong absorbing nature of perovskites. In our case of $\alpha \approx 2 \times 10^5\,\mathrm{cm}^{-2}$ about 63% of the generated carriers are located within the first 50 nm of the film. Due to the presence of a carrier gradient within the film, these carriers diffuse with time to generate a more homogeneous carrier distribution throughout the thickness of the film. Photons emitted by carriers at the bottom of the film might get re-absorbed by the material while traveling throughout the bulk, due to the small overlap of the absorption edge and the PL at the high energy side. This carrier diffusion and self-absorption process may thus lead to an attenuation of the PL at the high energy side and a small redshift. The diffusion time can be crudely estimated using $t = L^2/D$ (since we are only interested in diffusion into 1 direction) with common diffusion coefficients (D) for perovskites between 0.5 - 1.9 $\mathrm{cm}^2\mathrm{s}^{-1}$, and the film thicknesses (L) ($\approx$ 190 nm, 280 nm and 410 nm for MA, FA, and Cs respectively). With this, we get 0.2 - 0.7 ns for MA, 0.4 - 1.6 ns for FA, and 0.9 - 3.4 ns for Cs.[11–14] Since for FA and Cs these time scales reach the same order of magnitude as the described dynamics in the main text, we address the effect and the extent of the effect further below.

Diffusion and subsequent self-absorption cannot explain the observed initial blueshift with increasing carrier density and is thus not the dominant effect in describing the observed phenomena. In addition, diffusion and self-absorption would occur at low carrier density as well, while we observe minor to negligible red-shifting at low carrier density ($3 \times 10^{15}/9 \times 10^{15}$ cm^{-3}, see Figure 16) which is in stark contrast to the behavior at high carrier density (9×10^{17} cm^{-3}) where a pronounced redshift with time and faster decay at the high energy side is observed. For ease of comparison, both the high and low carrier density time-resolved semi-log false-color plots are given together in Figure 17, Figure 18, and Figure 19.

As stated, upon carrier density increase the PL blueshifts in a way that is compatible with band-filling. As a result, the PL emission will overlap more with the absorption spectrum,

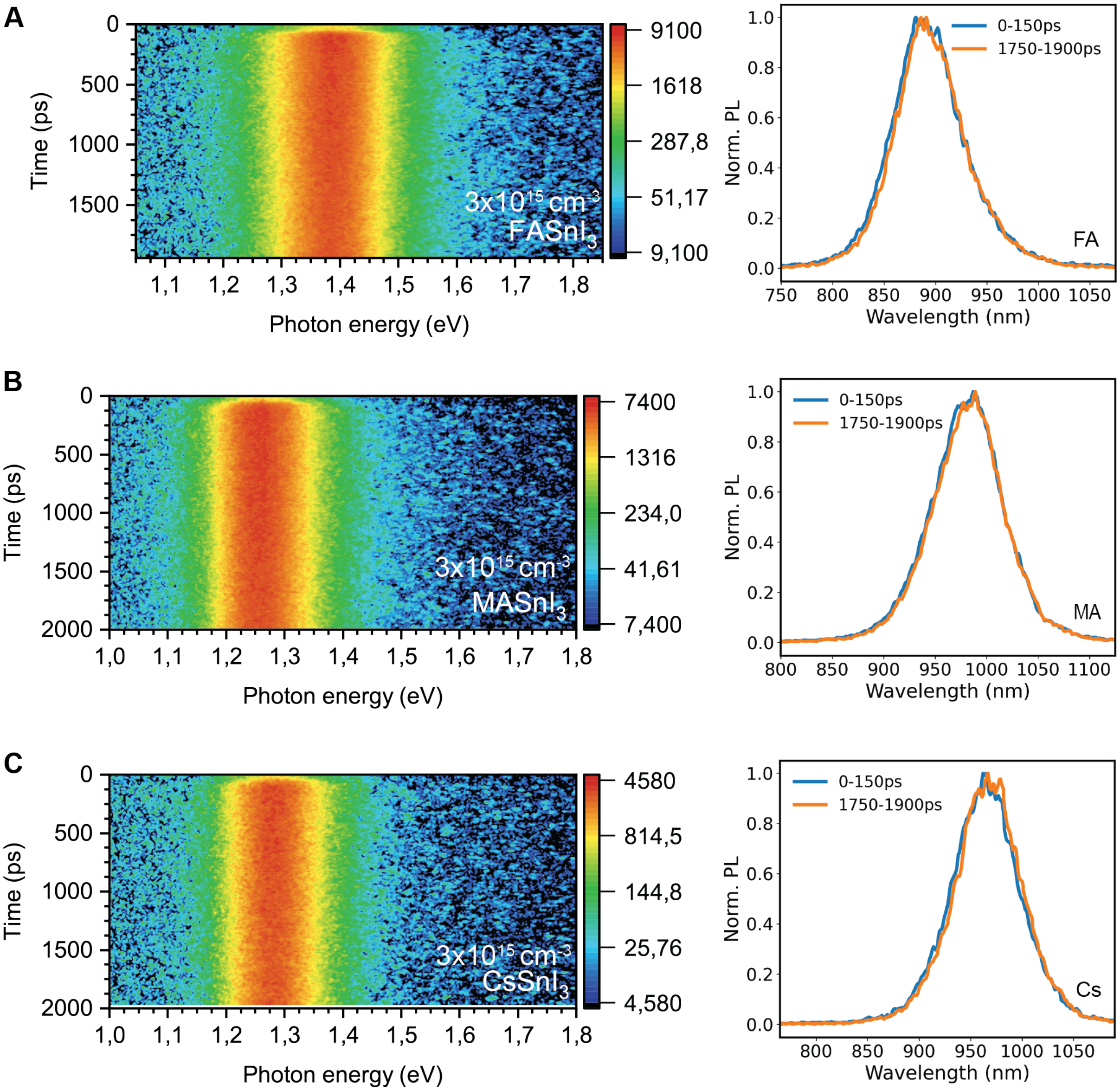

Supplementary Figure 16: (Left) False-color (logarithm of intensity) plot of the 7.5 nJcm^{-2} fluence (3×10^{15} cm^{-3} particle density) TR-PL spectrum of $FASnI_3$ (a), $MASnI_3$ (b), and $CsSnI_3$ (c). (Right) Energy-resolved spectra taken at specified times after initial excitation with 150 ps ROI for $FASnI_3$ (a), $MASnI_3$ (b), and $CsSnI_3$ (c).

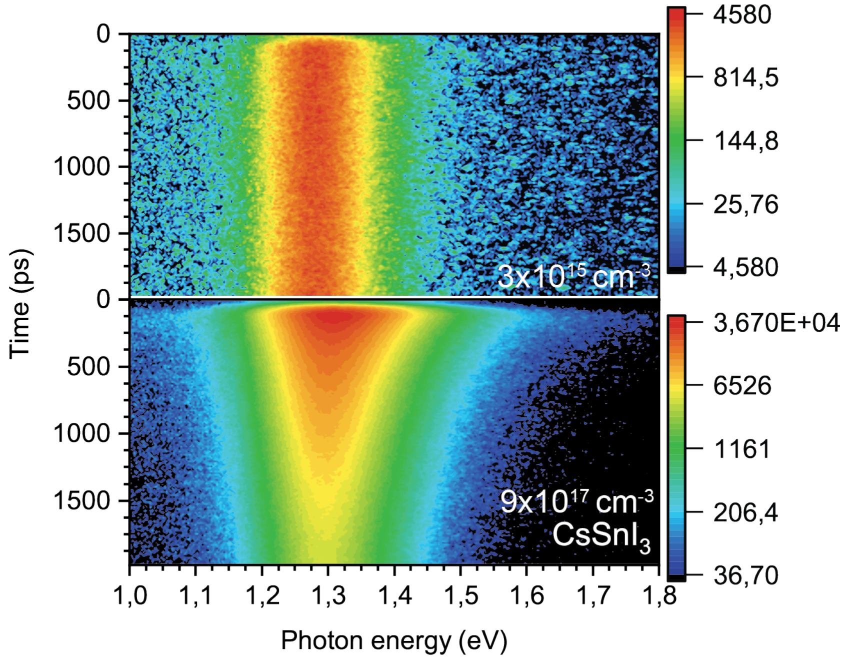

Supplementary Figure 17: False-color (logarithm of intensity) plot of the 7.5 nJcm^{-2} fluence (3×10^{15} cm^{-3} particle density) and 2.2 μJcm^{-2} fluence (9×10^{17} cm^{-3} particle density) TR-PL spectra of $CsSnI_3$.

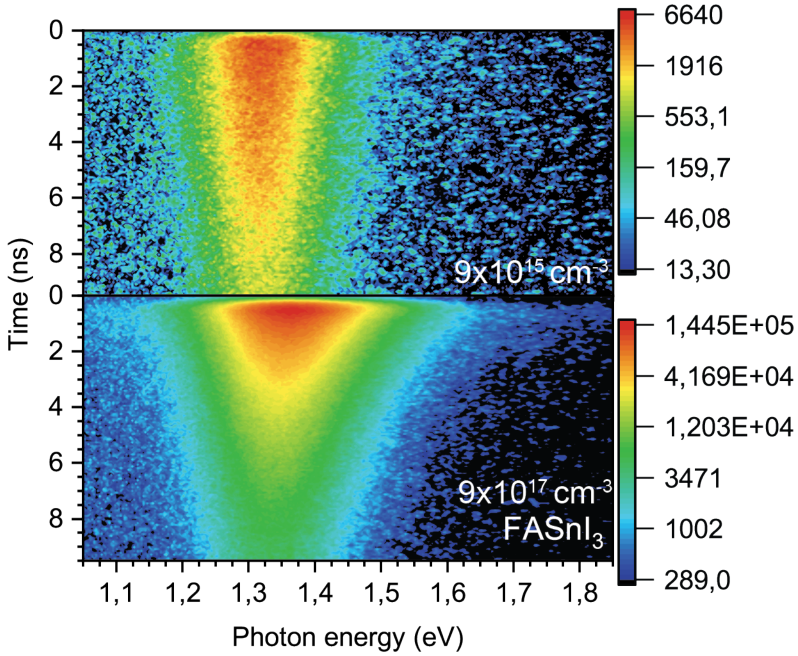

Supplementary Figure 18: False-color (logarithm of intensity) plot of the 22.3 nJcm^{-2} fluence (9×10^{15} cm^{-3} particle density) and 2.2 μJcm^{-2} fluence (9×10^{17} cm^{-3} particle density) TR-PL spectra of $FASnI_3$.

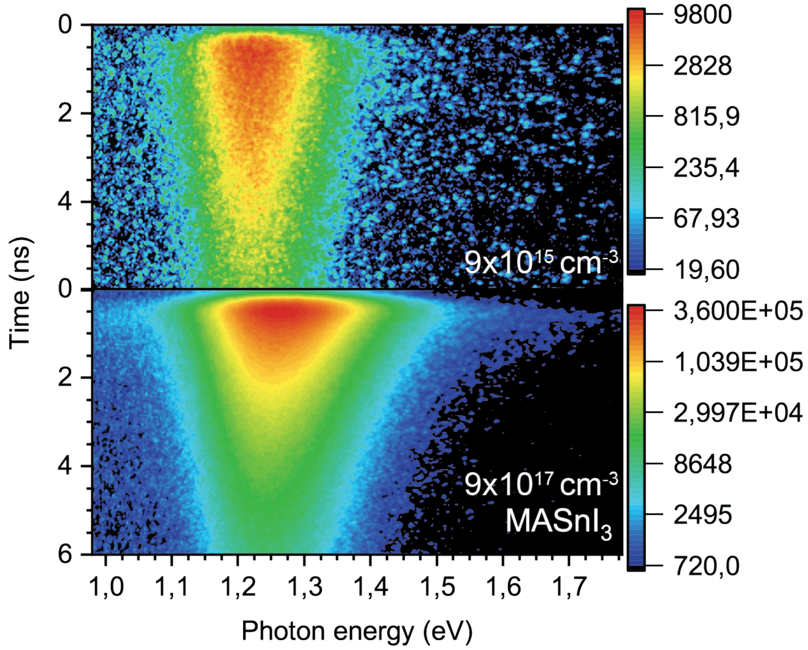

Supplementary Figure 19: False-color (logarithm of intensity) plot of the 22.3 nJcm^{-2} fluence (9×10^{15} cm^{-3} particle density) and 2.2 μJcm^{-2} fluence (9×10^{17} cm^{-3} particle density) TR-PL spectra of $MASnI_3$.

which could lead to a stronger self-absorption effect. To get an estimate of the extent of the effect in this case, we modeled the most extreme case of a fully diffused system, where the particle distribution throughout the thickness of the film is homogeneous, i.e. no carrier density gradient is present. This is done by attenuating the initial spectrum (assumed x = 0) by $I(x, \lambda) = I(0, \lambda)e^{-\alpha(\lambda)x}$, where x is the distance from the excitation spot. This is calculated at every distance x, where x is varied from 0 to the film thickness (L) in steps of L/999. The resulting attenuated spectrum is then taken as the average of those spectra, i.e. the final attenuated spectrum is:

$$I_{att} = \frac{\sum_{x \in I} I(0, \lambda)e^{-\alpha(\lambda)x}}{1000},$$

with I = {0, L/999, 2L/999, ..., 998L/999, L}. The results are given in Figure 20. For $FASnI_3$ and $MASnI_3$ (Figure 20a and 20b) it can be seen that the effect that diffusion has is minor and compared to the actual redshift measured negligible. For $CsSnI_3$, given in Figure 20c,

the calculated effect is non-negligible which is mainly due to the increased thickness of the $CsSnI_3$ film. However, still, the measured redshift is much stronger and for the calculation the most extreme diffusion case was taken which is questionable since only in a few exceptional cases diffusion lengths extending past 280 nm have been reported.[11,13] This is why also the case of 280 nm is given in Figure 20d. We therefore conclude that diffusion and subsequent self-absorption has a negligible to minor effect and is not the dominant effect explaining the observed phenomena.

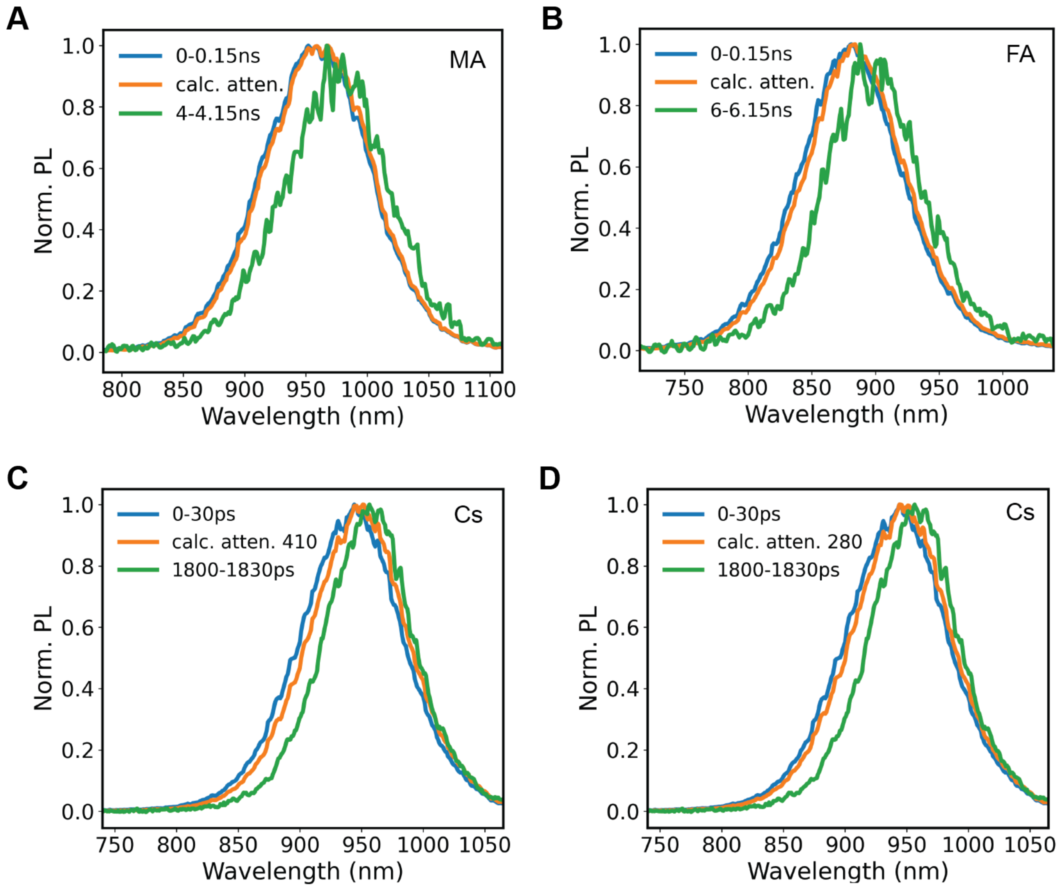

Supplementary Figure 20: Measured PL spectra directly after 2.2 μJcm^{-2} excitation (blue) and late in time (green) and the calculated attenuated spectrum by carrier diffusion and self-absorption (orange) for $MASnI_3$ (a), $FASnI_3$ (b), $CsSnI_3$ 410 nm (c), and $CsSnI_3$ 280 nm (d).

References

(1) Takahashi, Y.; Obara, R.; Lin, Z. Z.; Takahashi, Y.; Naito, T.; Inabe, T.; Ishibashi, S.; Terakura, K. Charge-transport in Tin-iodide Perovskite $CH_3NH_3SnI_3$: Origin of High Conductivity. *Dalton Transactions* **2011**, *40*, 5563–5568.

(2) Kahmann, S.; Nazarenko, O.; Shao, S.; Hordiichuk, O.; Kepenekian, M.; Even, J.; Kovalenko, M. V.; Blake, G. R.; Loi, M. A. Negative Thermal Quenching in $FASnI_3$ Perovskite Single Crystals and Thin Films. *ACS Energy Letters* **2020**, *5*, 2512–2519.

(3) Chung, I.; Song, J. H.; Im, J.; Androulakis, J.; Malliakas, C. D.; Li, H.; Freeman, A. J.; Kenney, J. T.; Kanatzidis, M. G. $CsSnI_3$: Semiconductor or Metal? High Electrical Conductivity and Strong Near-Infrared Photoluminescence from a Single Material. High Hole Mobility and Phase-transitions. *Journal of the American Chemical Society* **2012**, *134*, 8579–8587.

(4) Makula, P.; Pacia, M.; Macyk, W. How To Correctly Determine the Band Gap Energy of Modified Semiconductor Photocatalysts Based on UV–Vis Spectra. *The Journal of Physical Chemistry Letters* **2018**, *9*, 6814–6817.

(5) Noel, N. K.; Stranks, S. D.; Abate, A.; Wehrenfennig, C.; Guarnera, S.; Haghighirad, A. A.; Sadhanala, A.; Eperon, G. E.; Pathak, S. K.; Johnston, M. B. et al. Lead-free Organic-inorganic Tin Halide Perovskites for Photovoltaic Applications. *Energy and Environmental Science* **2014**, *7*, 3061–3068.

(6) Savill, K. J.; Klug, M. T.; Milot, R. L.; Snaith, H. J.; Herz, L. M. Charge-Carrier Cooling and Polarization Memory Loss in Formamidinium Tin Triiodide. *Journal of Physical Chemistry Letters* **2019**, *10*, 6038–6047.

(7) Konstantakou, M.; Stergiopoulos, T. A critical review on tin halide perovskite solar cells. *Journal of Materials Chemistry A* **2017**, *5*, 11518–11549.

(8) Filippetti, A.; Kahmann, S.; Caddeo, C.; Mattoni, A.; Saba, M.; Bosin, A.; Loi, M. A. Fundamentals of tin iodide perovskites: A promising route to highly efficient, lead-free solar cells. *Journal of Materials Chemistry A* **2021**, *9*, 11812–11826.

(9) Fang, H. H.; Adjokatse, S.; Shao, S.; Even, J.; Loi, M. A. Long-lived Hot-carrier Light Emission and Large Blue Shift in Formamidinium Tin Triiodide Perovskites. *Nature Communications* **2018**, *9*, 243.

(10) Manser, J. S.; Kamat, P. V. Band Filling with Free Charge Carriers in Organometal Halide Perovskites. *Nature Photonics* **2014**, *8*, 737–743.

(11) Wu, B.; Zhou, Y.; Xing, G.; Xu, Q.; Garces, H. F.; Solanki, A.; Goh, T. W.; Padture, N. P.; Sum, T. C. Long Minority-Carrier Diffusion Length and Low Surface-Recombination Velocity in Inorganic Lead-Free $CsSnI_3$ Perovskite Crystal for Solar Cells. *Advanced Functional Materials* **2017**, *27*, 1604818.

(12) Milot, R. L.; Klug, M. T.; Davies, C. L.; Wang, Z.; Kraus, H.; Snaith, H. J.; Johnston, M. B.; Herz, L. M. The Effects of Doping Density and Temperature on the Optoelectronic Properties of Formamidinium Tin Triiodide Thin Films. *Advanced Materials* **2018**, *30*, 1804506.

(13) Ma, L.; Hao, F.; Stoumpos, C. C.; Phelan, B. T.; Wasielewski, M. R.; Kanatzidis, M. G. Carrier Diffusion Lengths of over 500 nm in Lead-Free Perovskite CH3NH3SnI3 Films. *Journal of the American Chemical Society* **2016**, *138*, 14750–14755, PMID: 27750426.

(14) Ščajev, P.; Aleksiejunas, R.; Baronas, P.; Litvinas, D.; Kolenda, M.; Qin, C.; Fujihara, T.; Matsushima, T.; Adachi, C.; Juršėnas, S. Carrier Recombination and Diffusion in Wet-Cast Tin Iodide Perovskite Layers Under High Intensity Photoexcitation. *Journal of Physical Chemistry C* **2019**, *123*, 19275–19281.
